# Bi-directional associations between alcohol consumption and pain among non-manual workers: a random-intercept cross-lagged panel analysis in the British Whitehall II cohort study

**DOI:** 10.1093/alcalc/agag004

**Published:** 2026-01-31

**Authors:** Ziyi Zhao, Tea Lallukka, Tarani Chandola, Annie Britton

**Affiliations:** Institute of Epidemiology and Health Care, University College London, 1-19 Torrington Place, London WC1E 6BT, United Kingdom; Institute of Epidemiology and Health Care, University College London, 1-19 Torrington Place, London WC1E 6BT, United Kingdom; Department of Public Health, University of Helsinki, PL 20 (Tukholmankatu 8 B), Helsinki 00014, Finland; Department of Sociology, University of Hong Kong, 9/F, The Jockey Club Tower, Hong Kong, China; Institute of Epidemiology and Health Care, University College London, 1-19 Torrington Place, London WC1E 6BT, United Kingdom

**Keywords:** alcohol consumption, pain, retirement, socioeconomic position, longitudinal study

## Abstract

**Aims:**

The direction and temporality of the association between alcohol use and pain remain understudied among non-manual workers. This study investigated bidirectional associations between alcohol consumption and pain among current and retired non-manual workers, with exploratory subgroup analyses by retirement transition status, retirement age, and socioeconomic position (SEP).

**Methods:**

Survey data from Phases 7, 9, and 12 (2002–15) of the Whitehall II cohort study were analyzed (*n* = 5928, baseline mean age 60.7 years, 71% men). Alcohol consumption was assessed through self-reported intake converted to pure alcohol units. Pain severity was derived from musculoskeletal pain-site number and RAND-36 bodily pain measures, categorized as none, mild, or moderate/severe. Random-intercept cross-lagged panel models without equality constraints estimated within-person associations, with subgroup analyses by retirement transition and SEP.

**Results:**

At baseline, 23.1% reported above-moderate pain severity and 30.0% exceeded recommended limits (>14 units/week). During 14-year follow-up, 47.3% remained retired, 10.1% were employed, and 40.1% transitioned from employment to retirement. Elevated alcohol consumption was associated with increased pain severity among all participants (β_**P7 → P9**_ = 0.07, 95% confidence interval [CI]: 0.02–0.12; β_**P9 → P12**_ = 0.04, 95% CI: 0.00–0.08), with stronger effect among midlife retirees (β_**P9→P12**_ = 0.15, 95% CI: 0.04–0.25) and low-SEP participants (β_**P9→P12**_ = 0.22, 95% CI: 0.04–0.51). Pain was associated with alcohol consumption at earlier intervals, but associations attenuated subsequently.

**Conclusions:**

Elevated alcohol consumption might be associated with increased pain severity among non-manual workers, particularly midlife retirees and low-SEP individuals. Pain-to-alcohol associations were observed but were inconsistent across intervals.

## Teaser

This study examined bidirectional associations between alcohol and pain among non-manual workers. Within-person increases in alcohol consumption were associated with subsequent pain severity, particularly among midlife retirees and low-SEP individuals. Positive pain-to-alcohol associations were observed among retirees but attenuated over time.

## Introduction

Musculoskeletal disorders (MSDs) are the primary cause of non-cancer pain, affecting ~1.71 billion people worldwide and 18.8 million in the UK (Abraham et al. 2023). Chronic musculoskeletal pain (MSP) costs UK employers ~£21 032 per affected employee annually, with MSP-related absences accounting for nearly half of all sickness absence costs (Abraham et al. 2023). The working population represents a high-risk group for MSP due to sick-leave, premature exit from paid employment, and healthcare expenditure (Bonfiglioli et al. 2022).

Alcohol drinking is deeply embedded in British workplace culture, influenced by social norms and expectations, as well as occupational stress. An estimated 26%–36% of manual workers exhibit binge drinking behaviors, while 15%–27% of non-manual workers consume more than recommended weekly amounts (Emslie et al. 2002, Roche et al. 2015).

Alcohol and pain may be bidirectionally associated. Chronic problematic alcohol use can increase MSP likelihood through tissue damage and disrupted pain modulation, with stronger associations among males and those with substance use history (Ditre et al. 2019, Yeung et al. 2020, Roberts et al. 2021). Conversely, pain may increase alcohol consumption through stress-coping mechanisms, though evidence suggests that older adults with chronic multisite pain may reduce consumption (Brennan et al. 2005, Brennan and SooHoo 2013, LaRowe et al. 2020).

Existing studies have primarily examined alcohol–pain associations among manual workers (e.g. firefighters, military members), finding that frequent or problematic alcohol use may be associated with chronic multisite MSP compared to abstainers (Smith et al. 2006, Airila et al. 2014, Kim et al. 2017, Yepson et al. 2020). However, findings remain inconsistent due to heterogeneous assessments and methodologies. Non-manual workers have been less studied, despite evidence suggesting different risk patterns from manual workers: lower overall MSP prevalence but higher upper-body MSP due to prolonged sitting, awkward postures, and repetitive computer use (Akrouf et al. 2010, Zeverdegani et al. 2022, Li et al. 2024). Work-related stressors, such as long working hours and high job stress, may also increase problematic drinking among non-manual workers, particularly male and senior-level (Emslie et al. 2002, Marchand 2008, Heikkilä et al. 2013, Virtanen et al. 2015). Cross-sectional surveys have suggested a positive alcohol–pain relationships among non-manual workers, but prospective cohort studies examining bidirectional temporal dynamics are lacking (Akrouf et al. 2010, Li et al. 2024).

Retirement represents an essential transition point that may alter the alcohol–pain relationship. As individuals become unexposed to occupational stressors, lifestyle behaviors and musculoskeletal health may change (Kuerbis and Sacco 2012, Subas et al. 2018). Postretirement alcohol patterns show mixed findings: some retirees increase drinking due to reduced responsibilities, social isolation, or loss of daily structure, while others decrease consumption following age-related health conditions (Kuerbis and Sacco 2012). Similarly, work-related MSP may decrease with retirement, but age-related MSDs become more prevalent (Loeser 2010, Subas et al. 2018). Higher prevalence of problematic alcohol use has been observed among older retirees with chronic pain, yet prospective studies examining how retirement specifically modifies the alcohol–pain relationship remain scarce (LaRowe et al. 2024). Additionally, retirement age may influence this relationship, as midlife retirees are more likely to experience involuntary workforce exit and exhibit higher prevalence of both MSP and problematic alcohol use compared to those retiring later (Kaila-Kangas et al. 2009, Kuerbis and Sacco 2012).

Socioeconomic position (SEP) may also modify the alcohol–pain relationship among working populations. Higher SEP is associated with better working conditions, reduced job strain, and enhanced social support, which may promote healthier lifestyle choices and improved health outcomes (Hoven and Siegrist 2013). Research demonstrates that low-SEP workers may exhibit lower alcohol consumption yet experience higher rates of alcohol-related harm than those with higher SEP at equivalent consumption levels, a phenomenon known as the “alcohol-related harm paradox,” attributed to cumulative social disadvantages (Katikireddi et al. 2017, Khalatbari-Soltani and Blyth 2022). However, the moderating role of SEP on alcohol–pain associations remains understudied.

To our knowledge, no prospective study has examined the bidirectional temporal associations between alcohol consumption and pain among non-manual workers. This study applied random-intercept cross-lagged panel modeling (RI-CLPM) to investigate within-person associations between alcohol consumption and overall pain severity among current and retired non-manual workers. As exploratory analyses, we examined whether these associations were modified by retirement transition status and SEP, and whether associations among those experiencing retirement transition differed by retirement age.

## Materials and methods

### Study population

**Figure 1:**
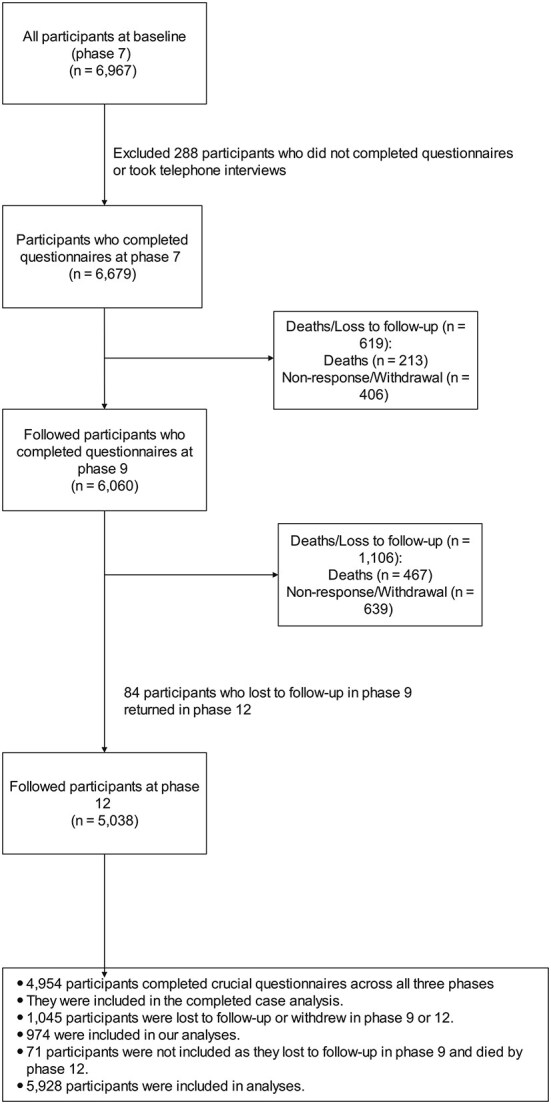
Flowchart.

The Whitehall II study is an ongoing, prospective cohort study collecting data from 10 308 British civil servants aged 35–55 years since 1985. Our study included Phases 7 (2002–04), 9 (2007–09), and 12 (2015–16), with response rates 71.6%, 72.3%, and 66.6%, respectively. The Phase 7 was chosen as the baseline of this study. These three phases were selected because they provided comprehensive assessments about alcohol consumption, MSP, bodily pain, and current employment status. Participants who did not complete the crucial questionnaires regarding these primary variables and other covariates or died by Phase 9 or Phase 12 were excluded (Fig. 1).

### Alcohol consumption

In a self-completed questionnaire, participants were asked about their drinking in the last week. Average weekly consumption of different alcoholic beverages was converted to pure alcohol units (1 unit = 8 g of pure alcohol) (UK Chief Medical Officers 2016). We also defined participants who consistently reported weekly alcohol use across three phases as consistent weekly drinkers. Analyses were conducted among this subgroup with more homogeneous drinking patterns to mitigate potential “sick-quitter” bias when assessing the alcohol-to-pain association. Additionally, prior studies examining pain-to-alcohol association have suggested that abstainers were unlikely to initiate alcohol use to cope with pain (Roberts et al. 2021).

### Pain

Overall pain severity was assessed using three components: MSP-site number from Nordic questionnaires covering back, upper extremities, and cervical regions (Kuorinka et al. 1987), bodily pain intensity, and pain interference from the RAND-36 questionnaire (Hays and Morales 2001). An equal-weighted composite score was calculated, with each component scored as 0 (no pain), 1 (mild or localized), or 2 (above-moderate or multisite). Participants were categorized as having no pain (0 points), mild pain (>0–3 points), or moderate/severe pain (>3–6 points). This approach captures anatomical distribution, subjective severity, and functional impact. These three dimensions may be associated with adverse health conditions (Castel et al. 2007, Butera et al. 2019, Pelletier et al. 2020). Detailed scoring methods are provided in Supplementary Material, along with the distribution of three individual pain metrics against the composite pain severity score (Supplementary Table S1).

### Baseline covariates

We included socio-demographic factors (age, sex, SEP, and current employment status), lifestyle factors (current smoking status, leisure-time physical activities [LTPA], body mass index, and sleep quality), mental well-being, morbidities, and analgesic use potentially associated with alcohol consumption and pain (Airila et al. 2014, Kirsch Micheletti et al. 2019).

SEP was categorized into high (administrative), intermediate (professional/executive), and low (clerical/support) groups based on the most recent employment grade for current employees and the final grade before retirement for retirees. Current employment status was classified as follows: retired, employed, unemployed, and others.

Lifestyle factors included obesity (body mass index [BMI] ≥30 kg/m^2^), weekly leisure-time physical activity (dichotomized using 2.5-h threshold), sleep quality (Jenkins sleep scale), and current smoking status (Jenkins et al. 1988, Hays and Morales 2001, Gimeno et al. 2009, NHS 2025a). Mental well-being was assessed using the five-item RAND-36 mental health (emotional well-being) score. Chronic morbidities included hypertension (≥140/90 mmHg), cardiovascular diseases (CVD), stroke, diabetes, respiratory illness, and cancer, ascertained through medical records and self-report (NHS 2025b). Analgesic use was based on prescribed medication.

### Statistical methods

RI-CLPM was implemented to evaluate the bi-directional association between alcohol consumption and overall pain severity. Unlike classic CLPM (Supplementary Fig. S1), RI-CLPM separates stable between-person differences from dynamic within-person processes through random intercepts (Fig. 1) (Hamaker et al. 2015). Given unequal intervals across phases (5 vs. 6–8 years), models without equality constraints are presented in primary analyses.

For ordinal pain outcomes, we used the latent response variable approach based on item-response theory and factor analytic frameworks (Muthén et al. 2024). In this approach, each observed categorical outcome is assumed to reflect an underlying continuous latent variable, with the categorical response determined by whether this latent variable exceeds specific threshold values. Measurement invariance for threshold values of ordinal pain outcomes was tested using scalar and approximate invariance constraints.

The relationships between these latent response variables were estimated through multivariate probit regression models. For the continuous pure-alcohol units, we implemented standardized log-linear regression. Primary estimates are reported as standardized regression coefficients. Marginal effects representing probability changes per standard deviation (SD) increase in alcohol consumption were calculated (see Supplementary Material for details). All analyses were conducted through Mplus 8.0, with script presented in Supplementary Material.

Full-information Bayesian estimation handled incomplete observations with non-informative priors (normal distribution with a mean of 0 and an infinite variance). Missing baseline covariates were imputed via multiple imputation with chained equation (MICE) with random forest algorithm, assuming data were missing at random (MAR). Ten imputed datasets were generated based on lower than 10% total missingness in baseline covariates. Pooled coefficient estimates and 95% confidence intervals were calculated using Rubin’s rules (Robin 1987).

### Subgroup analyses

Subgroup analyses were performed by retirement transition status across three phases and SEP at baseline. Working status changes were tracked across three phases to classify retirement transition status as follows: remained retired (consistently reported retired status), remained employed (consistently reported employed status), experienced retirement transition (transitioned from employment to retirement), and others (encompassed other transition patterns). Among participants experienced retirement transition, we further stratified by retirement age (<65 vs. ≥65 years), given that 60–65 years represents the typical retirement age for British civil servants. We used the retirement age as a proxy of retirement voluntariness because there were no questions regarding reasons of remaining employed or getting retired across three phases.

### Sensitivity analyses

Several sensitivity analyses tested robustness to missing data assumptions. Complete case analyses compared findings with the primary MAR-based RI-CLPM. Correlation tests evaluated whether baseline alcohol consumption and pain severity were associated with subsequent attrition. Linear latent growth models (LGMs) compared MAR (full-information maxmium likelihood [FIML] estimates) and missingness not at random (MNAR) (pattern mixture) assumptions for baseline effects on trajectories (see Supplementary Material; Staudt et al. 2022). Additionally, RI-CLPM was conducted with individual pain components to test robustness of the composite score.

## Results

**Figure 2:**
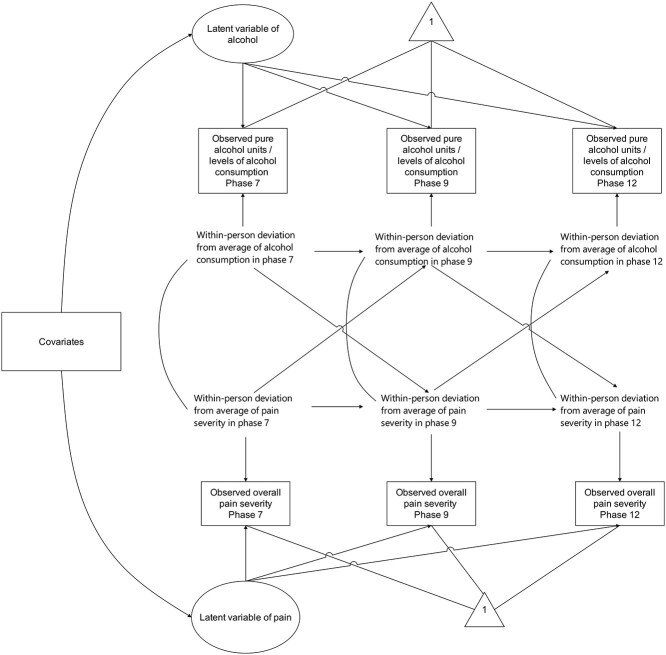
The framework of adjusted random-intercept cross-lagged panel model (RI-CLPM) in the study.

Of 6979 participants enrolled in Phase 7, 5928 (85.1%) were included in statistical analyses (Fig. 2). Two hundred eighty-eight (4.1%) participants were excluded for not completing crucial questionnaires. During 14-year follow-up, 680 (10.2%) participants died, and 1045 (15.6%) were lost to follow-up.

Table 1 shows distributions of longitudinal measurements. Median pure alcohol units decreased across phases. Pain-free participants decreased from 23.8% to 16.4%, while severe pain proportions remained stable (22%–23%). Employee proportions decreased from 52.2% to 9.2% as participants transitioned to retirement. Spaghetti plots of within-person deviations in alcohol consumption and latent pain severity, stratified by retirement transition and SEP, are shown in Supplementary Figs S2–S5.

**Table 1:** Sample size and descriptive statistics by phase and outcome variables.

Phase	Phase 7	Phase 9	Phase 12
*N*	5928	5928	5928
Age (mean [SD])	60.7 [5.8]	65.7 [5.9]	73.1 [5.9]
Sex (%)			
Female	1721 (29.0)	1721 (29.0)	1721 (29.0)
Pure alcohol units (median [IQR])	8.00 [2.00, 17.00]	7.00 [2.00, 15.00]	6.00 [1.00, 13.00]
Alcohol dependency indicator (%)			
No	5202 (87.8)	4611 (77.8)	-
Yes	581 (9.8)	531 (9.0)	-
Missing/loss to follow-up	145 (2.4)	786 (13.3)	-
Employment status (%)			
Employed	3097 (52.2)	1737 (29.3)	546 (9.2)
Retired	2497 (42.1)	3629 (61.2)	4263 (71.9)
Unemployed	251 (4.2)	195 (3.3)	210 (3.5)
Sick/disabled	83 (1.4)	28 (0.5)	12 (0.2)
Missing/loss to follow-up	0 (0.0)	339 (5.7)	897 (15.1)
RAND-36 bodily pain intensity (%)			
None	1580 (26.7)	1325 (22.4)	1051 (17.7)
Mild	3103 (52.3)	3076 (51.9)	2711 (45.7)
Moderate or higher	1187 (20.0)	1139 (19.2)	1228 (20.7)
Missing/loss to follow-up	58 (1.0)	688 (6.5)	938 (15.8)
RAND-36 bodily pain interference (%)			
None	3852 (65.0)	3533 (59.6)	2859 (48.2)
Mild	1369 (23.1)	1370 (23.1)	1348 (22.7)
Moderate or higher	646 (10.9)	436 (7.4)	518 (8.7)
Missing/loss to follow-up	61 (1.0)	589 (9.9)	1203 (20.3)
Recent MSP site number (%)			
None	3499 (59.0)	3272 (55.2)	2842 (47.9)
1–2 sites	2036 (34.3)	1929 (32.5)	1787 (30.1)
≥3 sites	327 (5.5)	319 (5.4)	280 (4.7)
Missing/loss to follow-up	66 (1.1)	408 (6.9)	1019 (17.2)
Overall pain severity (%)			
None	1381 (23.3)	1156 (19.5)	961 (16.4)
Mild pain	3140 (52.7)	3060 (51.9)	2595 (43.8)
Severe pain	1369 (23.1)	1308 (22.1)	1391 (23.5)
Missing/loss to follow-up	54 (0.9)	404 (6.8)	981 (16.5)

IQR, interquartile; MSP, musculoskeletal pain; SD, standard deviation.

- : alcohol dependency questionnaires were not available in Phase 12.

Baseline characteristics by pain severity are shown in Supplementary Table S2. Median pure-alcohol units decreased as pain severity increased. There were higher proportions of female participants and low-SEP individuals among participants with above-moderate pain severity. Furthermore, participant with more severe pain appears to have higher prevalence of unhealthy lifestyles (e.g. obesity, low LTPA, and sleep disturbance), chronic morbidities (e.g. hypertension, CVD, history of respiratory illness), and prescribed analgesic use.

Total missingness in baseline covariates was 7.9%, with most individual covariates below 5% (Supplementary Table S3). Combined loss to follow-up and deaths exceeds 20% at Phases 9 and 12 (Supplementary Table S4).

### Primary analysis

#### Autoregressive associations

Within-person deviations in alcohol consumption at Phase 7 were associated with elevated consumption at Phase 9 (β_adjusted_ = 0.28, 95% confidence interval [CI]: 0.24–0.32), but deviations at Phase 9 were followed by reduced consumption at Phase 12 (β_adjusted_ = −0.20, 95% CI:−0.32–0.10) (Table 2). Overall pain severity showed consistent positive autoregressive associations across both intervals (β_P7→P9_ = 0.10, 95% CI: 0.03–0.17; β_P9→P12_ = 0.11, 95% CI: 0.04–0.18).

**Table 2:** Pooled association between pure alcohol consumption and overall pain severity using crude and fully-adjusted random-intercept cross-lagged panel model (RI-CLPM) without equality constraints on autoregressive and cross-lagged pathways.

	Subsequent alcohol outcomes^a^	Subsequent pain outcomes^b^
Prior-phase variables	Standardized beta (95% CI)	Standardized beta (95% CI)
**Model 1: Crude model**		
**Among all participants (*n* = 5928)**		
Pure alcohol units (Phase 7)	0.28 (0.24, 0.33)	0.06 (0.01, 0.12)
Pure alcohol units (Phase 9)	−0.36 (−0.54, −0.21)	0.03 (−0.01, 0.08)
Overall pain severity (Phase 7)	0.04 (0.00, 0.08)	0.09 (0.02, 0.16)
Overall pain severity (Phase 9)	0.04 (−0.06, 0.14)	0.08 (0.01, 0.16)
**Among consistent weekly drinkers (*n* = 3350)**		
Pure alcohol units (Phase 7)	0.24 (0.18, 0.29)	0.09 (0.02, 0.16)
Pure alcohol units (Phase 9)	−0.16 (−0.29, −0.04)	0.03 (−0.03, 0.08)
Overall pain severity (Phase 7)	0.03 (−0.02, 0.16)	0.07 (−0.02, 0.15)
Overall pain severity (Phase 9)	0.04 (−0.06, 0.14)	0.07 (−0.02, 0.15)
**Model 2: Adjusted model** ^**c**^		
**Among all participants (*n* = 5928)**		
Pure alcohol units (Phase 7)	0.28 (0.24, 0,32)	0.07 (0.02, 0.12)
Pure alcohol units (Phase 9)	−0.20 (−0.32, −0.10)	0.04 (0.00, 0.08)
Overall pain severity (Phase 7)	0.05 (0.01, 0.08)	0.10 (0.03, 0.17)
Overall pain severity (Phase 9)	0.03 (−0.05, 0.11)	0.11 (0.04, 0.18)
**Among consistent weekly drinkers (*n* = 3350)**		
Pure alcohol units (Phase 7)	0.24 (0.19, 0.29)	0.08 (0.01, 0.14)
Pure alcohol units (Phase 9)	−0.10 (−0.20, 0.00)	0.03 (−0.02, 0.08)
Overall pain severity (Phase 7)	0.04 (−0.01, 0.08)	0.07 (−0.02, 0.15)
Overall pain severity (Phase 9)	0.03 (−0.06, 0.12)	0.07 (−001, 0.16)

Bayesian posterior-probability checking (PPC) *P*-values for crude and adjusted models are .000 and .321, respectively; PPC *P*-values for two models among consistent weekly drinkers are .000 and .297.

^a^Subsequent alcohol outcome: within-person autoregressive associations between alcohol at Phase 7 and alcohol at Phase 9, and association between alcohol at Phase 9 and alcohol at Phase12; within-person cross-lagged association between pain at Phase 7 and alcohol at Phase 9, and association between pain at Phase 9 and alcohol at Phase 12.

^b^Subsequent pain outcome: within-person autoregressive associations between pain at Phase 7 and pain at Phase 9, and association between pain at Phase 9 and pain at Phase12; within-person cross-lagged association between alcohol at Phase 7 and pain at Phase 9, and association between alcohol at Phase 9 and pain at Phase 12.

^c^Adjusted model: adjusted potential baseline covariates, including socio-demographic (age, sex, SEP, employment status), lifestyle (BMI, smoking, sleep quality, leisure-time physical activities), mental well-being, chronic morbidities (hypertension, cardiovascular diseases, stroke, diabetes, respiratory illness, cancer), and prescribed analgesic use.

#### Alcohol-to-pain

Within-person increases in pure alcohol intake at Phase 7 predicted increased pain severity at Phase 9 ($\mathrm{\beta}$ = 0.06, 95% CI: 0.01–0.12), with similar results after covariates adjustment ($\mathrm{\beta}$ = 0.07, 95% CI: 0.02–0.12). The association from Phases 9 to 12 was weaker in both crude ($\mathrm{\beta}$ = 0.03, 95% CI: −0.01–0.08) and adjusted models ($\mathrm{\beta}$ = 0.04, 95% CI: 0.00–0.08). Among weekly drinkers, only association from Phases 7 to 9 remained significant (β = 0.08, 95% CI: 0.01–0.14). Expressed as marginal effects, every SD increases (13.0 units) in pure alcohol units at Phases 7 and 9 was associated with 17.7% and 20.2% relative increases in above-moderate pain-severity probability at subsequent phases (Supplementary Fig. S6).

#### Pain-to-alcohol

The pain-to-alcohol associations were inconsistent across intervals. Within-person increases in latent pain severity at Phase 7 predicted increased pure alcohol consumption at Phase 9 (β_adjusted_ = 0.05, 95% CI: 0.01–0.08), but no association was observed from Phases 9 to 12 (β_adjusted_ = 0.03, 95% CI: −0.05–0.11).

### Subgroup analysis

#### By retirement transition status

Among retired participants (*n* = 2311), within-person deviations of alcohol consumption at Phase 9 predicted increased pain severity at Phase 12 (β = 0.07, 95% CI: 0.01–0.14), corresponding to a 35.0% increase in above-moderate-pain likelihood per SD increase (Supplementary Fig. S7), though association from Phases 7 to 9 was non-significant (Table 3). Deviations in latent pain severity at Phase 7 were associated with increased alcohol consumption at Phase 9 (β = 0.07, 95% CI: 0.02–0 .13), translating to a 10.9% increase in pure alcohol intake (Supplementary Fig. S8), but no association was observed at later interval.

**Table 3:** Pooled associations between pure alcohol consumption and overall pain severity through fully-adjusted RI-CLPM without equality constraints on autoregressive and cross-lagged pathways, stratified by retirement transition status.

	Subsequent alcohol outcomes^a^	Subsequent pain outcomes^b^
Prior-phase variables	Standardized beta (95% CI)	Standardized beta (95% CI)
**Among participants remaining retired (*n* = 2311)**		
Pure alcohol units (Phase 7)	0.31 (0.25, 0.36)	0.05 (−0.04, 0.13)
Pure alcohol units (Phase 9)	−0.24 (−0.47, −0.06)	0.07 (0.01, 0.14)
Overall pain severity (Phase 7)	0.07 (0.02, 0.13)	0.05 (−0.06, 0.15)
Overall pain severity (Phase 9)	0.03 (−0.15, 0.20)	0.09 (−0.02, 0.20)
**Among participants experiencing retirement transition (*n* = 2378)**		
Pure alcohol units (Phase 7)	0.22 (0.13, 0.30)	0.07 (0.00, 0.14)
Pure alcohol units (Phase 9)	−0.12 (−0.30, 0.04)	0.02 (−0.05, 0.10)
Overall pain severity (Phase 7)	0.02 (−0.05, 0.09)	0.10 (0.00, 0.21)
Overall pain severity (Phase 9)	0.05 (−0.04, 0.13)	0.09 (−0.02, 0.19)
**Among participants remaining employed (*n* = 597)**		
Pure alcohol units (Phase 7)	0.33 (0.18, 0.48)	0.07 (−0.07, 0.21)
Pure alcohol units (Phase 9)	−0.03 (−0.70, 0.34)	−0.08 (−0.23, 0.09)
Overall pain severity (Phase 7)	−0.11 (−0.24, 0.03)	0.11 (−0.10, 0.33)
Overall pain severity (Phase 9)	−0.08 (−0.30, 0.14)	0.13 (−0.10, 0.35)

The Bayesian PPC *P*-value for analyses among retirees, transitioning participants, employees are .251, .292, and .233.

^a^Subsequent alcohol outcome: within-person autoregressive associations between alcohol at Phase 7 and alcohol at Phase 9, and association between alcohol at Phase 9 and alcohol at Phase 12; within-person cross-lagged association between pain at Phase 7 and alcohol at Phase 9, and association between pain at Phase 9 and alcohol at Phase 12.

^b^Subsequent pain outcome: within-person autoregressive associations between pain at Phase 7 and pain at Phase 9, and association between pain at Phase 9 and pain at Phase 12; within-person cross-lagged association between alcohol at Phase 7 and pain at Phase 9, and association between alcohol at Phase 9 and pain at Phase 12.

Among participants experiencing retirement transition (*n* = 2378), only within-person alcohol-to-pain association from Phases 7 to 9 reached borderline significance (β = 0.07, 95% CI: 0.09–0.14), corresponding to a 19.9% increase in above-moderate-pain likelihood per SD increase (Supplementary Fig. S9). No association was observed at later interval (β = 0.02, 95% CI: −0.05–0.10) or in the reverse direction.

#### By retirement age

Among midlife retirees (*n* = 1021), alcohol consumption at Phase 9 was associated with increased pain severity at Phase 12 (β = 0.15, 95% CI: 0.04–0.25), corresponding to a 49.3% increase in above-moderate-pain probability per SD increase (Supplementary Fig. S10). The association from Phases 7 to 9 could not be confirmed (β = 0.06, 95% CI: -0.05–0.18) (Supplementary Table S5).

#### By socioeconomic position

Among low-SEP participants (*n* = 614), within-person deviations in alcohol consumption at Phase 9 were associated with increased pain severity at Phase 12 (β = 0.22, 95% CI: 0.04–0.51), corresponding to a 65.7% increase in above-moderate-pain probability per SD increase (Supplementary Fig. S11), but no association was observed at the earlier interval (Supplementary Table S6). Among high-SEP participants (*n* = 2739), only association from Phases 7 to 9 reached borderline significance (β = 0.06, 95% CI: 0.00–0.13), corresponding to a 13.3% increase per SD increase (Supplementary Fig. S12). The association at later interval could not be confirmed.

### Sensitivity analyses under different missingness assumptions

#### Complete case analysis

Among participants with complete observations (*n* = 4249), within-person associations were similar with the full-sample MAR analysis, but effect estimates of associations from Phases 9 to 12 were attenuated (Supplementary Table S7).

#### Correlation test

Both baseline alcohol consumption and pain severity significantly correlated with loss to follow-up or death (all *P* < .05; Supplementary Table S8), suggesting the data may not be missing completely at random.

#### Pattern mixture models

Six distinct missing data patterns were identified and categorized into complete data (71%, *n* = 4954), partial data (21%, *n* = 1474), and no follow-up (8%, *n* = 251) (Supplementary Table S9).

Baseline pain severity was not significantly associated with alcohol consumption trajectories under any assumptions (Supplementary Table S10). At Phase 12, estimates for severe versus no pain ranged from −0.550 (MAR) to −0.579 (MNAR-complete case), with maximum variation of 0.067 units (13%) and all confidence intervals including zero. Results were similarly consistent for mild pain and at Phase 9.

Associations between baseline alcohol and pain trajectories varied by assumptions, particularly for excessive drinkers (Supplementary Table S11). For excessive drinkers at Phase 12, estimates varied 4.5-fold (0.214 to 0.970) depending on assumptions. Though MAR models suggested no association, two of three MNAR models indicated significant positive associations between excessive drinking and pain progression.

### Sensitivity analyses with separate pain metrics

Within-person associations between alcohol and individual pain components paralleled composite score findings (Supplementary Tables S12–S14). Within-person deviation of alcohol consumption predicted greater subsequent bodily pain intensity (β_P7 → P9_ = 0.06, 95% CI: 0.01–0.11; β_P9→P12_ = 0.03, 95% CI: 0.00–0.08), but associations between alcohol consumption and bodily pain interference as well as MSP-site number were inconsistent across intervals. Only within-person increase in bodily pain intensity predicted increased pure alcohol consumption from Phases 7 to 9 (β_P7→P9_ = 0.05, 95% CI: 0.01–0.09), but such association could not be confirmed at the later interval.

## Discussion

Our study examined the temporality and direction of the association between alcohol consumption and overall pain severity through RI-CLPM among non-manual workers. In the primary analysis, within-person increase of alcohol consumption was associated with elevated subsequent pain severity, though effect estimates attenuated across intervals. Conversely, within-person increase in overall pain severity was associated with increased pure alcohol consumption at the earlier intervals, with null associations at the later interval. Subgroup analyses revealed effect modification by retirement status and SEP, with stronger alcohol-to-pain associations among retired participants, midlife retirees, and low-SEP individuals at the later interval, and among those experiencing retirement transition and high-SEP individuals at the earlier interval. Pain-to-alcohol associations were observed only among retirees at the earlier interval.

This study offers several methodological advantages over previous research. Unlike prior studies using LGMs or classic CLPM, RI-CLPM captures dynamic bidirectional associations while separating within-person effects from stable between-person differences (Brennan and SooHoo 2013, Airila et al. 2014, Yeung et al. 2020, Roberts et al. 2021).

### Alcohol-to-pain associations

The positive within-person associations between alcohol consumption and subsequent pain severity are consistent with reviews and longitudinal studies demonstrating the harmful effects of excessive alcohol consumption on chronic pain (Ditre et al. 2019, Roberts et al. 2021). The observed significance may partly reflect the predominantly male composition of our sample (70%), as previous research demonstrates that male individuals with problematic alcohol use exhibit higher pain severity (Yeung et al. 2020). Our prospective findings provide longitudinal evidence supporting associations that were previously identified primarily through cross-sectional surveys among non-manual workers (Akrouf et al. 2010, Li et al. 2024).

Despite consistent positive associations, effect estimates attenuated toward the null at the later interval. This attenuation may reflect healthy survivor bias. Attenuated effect estimates in complete case analysis and significant correlations between baseline alcohol consumption and pain severity with subsequent loss to follow-up and deaths questioned the robustness of findings under MCAR and MAR assumptions. Pattern mixture analyses under MNAR assumptions revealed great differences in the effect of excessive alcohol consumption on pain severity compared to MAR-based models, potentially indicating that individuals with excessive drinking patterns who might develop severe pain exited the cohort through death or loss to follow-up.

Subgroup analyses revealed stronger alcohol-to-pain associations among retired participants and midlife retirees at the later interval, potentially suggesting increased vulnerability in these groups. This may partly reflect increased vulnerability to alcohol-related harm among older adults (LaRowe et al. 2024, Ortolá et al. 2024), and retirement-specific factors, such as social isolation, loss of daily structure, and reduced employer health monitoring, that may promote alcohol use as a coping mechanism (Kuerbis and Sacco 2012, Bamberger 2015). The strong association among transitioning employees and midlife retirees might reflect higher rates of involuntary workforce exits in this group, who may experience elevated chronic pain conditions compared to their working counterparts (Kaila-Kangas et al. 2009). However, these mechanisms would require direct measurement to test empirically.

Among low-SEP individuals, a strong positive association was observed at the later interval, but interpretation is limited by wide confidence intervals (Supplementary Table S6) and 40% incomplete observations (Supplementary Table S15). Baseline characteristics of low-SEP participants matched the social disadvantages described in the “alcohol-related harm paradox,” such as higher proportions of adverse health indicators (obesity, current smoking, hypertension, CVD) and lower mental wellbeing compared to higher SEP groups (Supplementary Table S16), but limited power undermined the robustness of this association (Katikireddi et al. 2017).

Among high-SEP participants, positive associations were observed at the earlier interval but attenuated at the later interval, consistent with the pattern observed in primary analyses. This pattern may reflect higher baseline alcohol consumption and greater proportions of above-moderate drinkers among high-SEP participants (Supplementary Table S16).

### Pain-to-alcohol associations

We observed positive pain-to-alcohol associations at the earlier interval among the full sample and retirees. Component-specific sensitivity analyses revealed that only increased bodily pain intensity at Phase 7 predicted increased alcohol consumption at Phase 9, potentially driving findings for the composite overall-pain-severity score. These findings are consistent with studies demonstrating higher prevalence of problematic alcohol use among older and retired populations with chronic pain, possibly reflecting elevated alcohol consumption in response to pain-related stress (LaRowe et al. 2024). Severe pain and high interference can contribute to psychological distress among retirees, who face retirement-related stressors that may lower coping abilities and subsequently increase alcohol consumption (Ditre et al. 2019, Shoushtari-Moghaddam et al. 2024). Although our analyses adjusted emotional well-being that correlated with alcohol consumption and pain (Supplementary Table S17), we lacked assessment of pain-related stress or drinking motives across phases, limiting our ability to empirically test this stress-coping pathway.

The attenuated pain-to-alcohol association at later interval might still reflect healthy survivor bias, though pattern mixture analyses showed minimal differences between MAR and MNAR models. This null finding may be caused by pain measurement limitations: our MSP assessment captured only upper-body pain sites and may inadequately assess pain among older retirees, for whom lower limb pain is prevalent. Although bodily pain intensity and interference were included to offset this anatomical restriction, the most severe cases with widespread pain may still be misclassified. These individuals may be most likely to both increase alcohol consumption in response to pain and drop out, meaning our sensitivity analyses might not detect MNAR bias due to truncation of the pain measure.

### Limitations

First, participants were predominantly midlife-to-older British White civil servants, limiting generalizability. Second, our data lacked time-variant assessment of problematic alcohol use, though above-moderate weekly alcohol consumption may also indicate drinking behaviors at risk, especially among older individuals.

Third, subgroup analyses were exploratory. Retirement transition was assessed through tracking the change of self-reported employment status. SEP was assessed using preretirement occupational grade only. We lacked questionnaires assessing retirement reasons for those midlife transitioning participants. Multi-dimensional and dynamic assessments of retirement transitions, retirement voluntariness, and SEP are needed to confirm these findings.

Fourth, though we acquired poor model fit from analyses with scalar measurement invariance for ordinal pain outcomes, acceptable model fit was achieved under approximate invariance with relaxed Bayesian priors, suggesting that threshold deviations across phases were modest (Supplementary Table S18). Such drift may reflect increased pain sensitivity due to aging or prolonged exposure to chronic pain (Gibson and Farrell 2004, Woolf 2011).

Fifth, alcohol consumption was assessed for the prior week, whereas pain covered the prior month, creating a temporal mismatch. However, the 1-month pain window captures more stable pain experiences rather than episodic pain, while the weekly alcohol measure provides a snapshot of recent consumption with minimal recall bias. Given that our study examined associations across multi-year intervals, the difference between 7-day and 30-day recall windows is relatively minor compared to the broader temporal dynamics investigated. Finally, only three phases over 14 years with unequal intervals limited within-phase variability assessment, though repeated measurements represent an advantage over existing cross-sectional studies.

## Conclusions

This study provides prospective evidence for potential bidirectional associations between alcohol consumption and pain among non-manual workers. Within-person increases in alcohol consumption might be associated with subsequent elevations in pain severity, particularly among midlife retirees and low-SEP individuals. Within-person elevations in pain severity may be associated with subsequent increases in alcohol consumption among retirees. However, attenuated and inconsistent effect estimates across intervals preclude firm causal conclusions. Future studies with more frequent assessments, equal-interval follow-up, time-variant problematic alcohol measures, and detailed retirement and SEP questionnaires are warranted to verify these exploratory findings.

## Supplementary Material

Supplementary_materials_agag004

## Data Availability

The Whitehall II data are available through UK Data Service and requests to the study team. Researchers wishing to access the data can apply for access through following websites: https://www.ucl.ac.uk/brain-sciences/psychiatry/our-research/mental-health-older-people/whitehall-ii/data-sharing.
