# Supplementary material for: Bi-directional associations between alcohol consumption and pain among non-manual workers: a random-intercept cross-lagged panel analysis in the British Whitehall II cohort study
Source: Alcohol Alcohol. 2026 Jan 31;61(2):agag004. doi: 10.1093/alcalc/agag004 (PMC12860205; doi:10.1093/alcalc/agag004)
Supplement: Supplementary_materials_agag004 [file supplementary_materials_agag004.docx]

**Supplementary material**

**Calculation of overall pain severity score**

The distribution of MSP sites was assessed using standard Nordic questionnaires, including back, upper extremities, and cervical regions (Kuorinka et al. 1987). Participants reported pain experiences from the past year and previous two weeks. The total number of recent MSP sites recurring or lasting more than one week was calculated, and participants were categorized as having none (0 sites), localized pain (1–2 sites), or multisite pain (>2 sites).

Bodily pain was assessed using two items from the RAND-36 questionnaire measuring pain intensity and pain interference with daily activities during the preceding four weeks (Hays and and Morales 2001). Pain intensity was rated on a six-point scale (none, very mild, mild, moderate, severe, very severe). Pain interference was rated on a five-point scale (not at all, a little bit, moderately, quite a bit, extremely). Both pain indicators were categorized into three levels: none, mild, and moderate or higher.

An overall pain severity score was constructed using equal-weighted sum scores from three pain components: pain sites (none = 0, localized = 1, multisite = 2), bodily pain intensity (none = 0, mild = 1, moderate or severe = 2), and bodily pain interference (none = 0, mild = 1, moderate or higher = 2). Equal weighting was applied to these three components because they assess pain severity from distinct dimensions and are considered to have equivalent clinical importance. Each component was scored on a three-point scale (0–2), and the total pain severity score ranged from 0 to 6. This composite score was then categorized into three levels: none (score = 0), mild (score = 1–2), and above-moderate (score = 3–6) pain severity.

The weighted-sum approach could be referred to Brief Pain Inventory (BPI), though our pain items were simpler than BPI (Poquet and Lin, 2016). Three pain indicators were assigned with equal weight for clinical rationales: each captures a different dimension of the pain experience: anatomical distribution (number of MSP sites), subjective severity (intensity), and impact on physical functions (interference). This equal-weight approach ensures that participants experiencing any one of the following are classified as having moderate or severe pain: multisite pain, moderate-to-severe intensity, or moderate-to-severe interference. This classification reflects clinical evidence that any of these features is associated with adverse prognostic outcomes (Castel et al. 2007; Butera et al. 2019; Pelletier et al. 2020).

**Table S1**. The distribution of three pain metrics across three levels of overall pain severity.

| **Overall pain severity score at phase 7** | | | | |
| --- | --- | --- | --- | --- |
|  | **None** | **Mild/localized pain** | **Moderate or severe/multisite pain** | **Missing** |
| N | 1410 | 3140 | 1369 | 9 |
| Number of recent MSP sites |  |  |  |  |
| None | 1410 (100.0) | 1765 (56.2) | 390 (28.5) | 0 (0.0) |
| Localized (1-2 sites) | 0 (0.0) | 1375 (43.8) | 652 (47.6) | 0 (0.0) |
| Multisite (>2 sites) | 0 (0.0) | 0 (0.0) | 327 (23.9) | 0 (0.0) |
| Missing | 0 (0.0) | 0 (0.0) | 0 (0.0) | 9 (100.0) |
| RAND-36 Bodily Pain Intensity |  |  |  |  |
| None | 1410 (100.0) | 214 (6.8) | 7 (0.5) | 0 (0.0) |
| Mild | 0 (0.0) | 2926 (93.2) | 175 (12.8) | 0 (0.0) |
| Moderate or severe | 0 (0.0) | 0 (0.0) | 1187 (86.7) | 0 (0.0) |
| Missing | 0 (0.0) | 0 (0.0) | 0 (0.0) | 9 (100.0) |
| RAND-36 Bodily Pain Interference |  |  |  |  |
| None | 1410 (100.0) | 2256 (71.8) | 234 (17.1) | 0 (0.0) |
| Mild | 0 (0.0) | 884 (28.2) | 485 (35.4) | 0 (0.0) |
| Moderate or higher | 0 (0.0) | 0 (0.0) | 650 (47.5) | 0 (0.0) |
| Missing | 0 (0.0) | 0 (0.0) | 0 (0.0) | 9 (100.0) |

**Reference**:

Butera, Katie A., et al. (2019), 'The impact of multisite pain on functional outcomes in older

adults: biopsychosocial considerations', *Journal of Pain Research,* 12 (null), 1115-25.

Castel, Liana D., et al. (2007), 'Hazards for Pain Severity and Pain Interference with Daily

Living, with Exploration of Brief Pain Inventory Cutpoints, Among Women with

Metastatic Breast Cancer', *Journal of Pain and Symptom Management,* 34 (4), 380-92.

Hays, Ron D. and and Morales, Leo S. (2001), 'The RAND-36 measure of health-related quality

of life', *Annals of Medicine,* 33 (5), 350-57.

Kuorinka, I., et al. (1987), 'Standardised Nordic questionnaires for the analysis of

musculoskeletal symptoms', *Appl Ergon,* 18 (3), 233-7.

Pelletier, René, et al. (2020), 'Pain interference may be an important link between pain severity, impairment, and self-reported disability in participants with wrist/hand pain', *Journal of*  *Hand Therapy,* 33 (4), 562-70.e1.

Poquet, Nolwenn and Lin, Christine (2016), 'The Brief Pain Inventory (BPI)', *Journal of Physiotherapy,* 62 (1), 52.

**Equations of probability calculation in the probit regression**

Probability translation in the (three-level) ordinal probit regression model:

$$P\left( u=0 \right|x)=F(t_{1}-b*x)$$

$$P\left( u=1 \right| x)=F\left( t_{2}-b*x \right)-F(t_{1}-b*x)$$

$$P\left( u=2 \right| x)=F(-t_{2}+b*x)$$

where *u* stands for the subsequent pain outcomes in this case (0 indicates no pain, 1 indicates mild pain, 2 indicates severe pain). $t_{1}$ and $t_{2}$ stand for threshold values of latent response variable for subsequent pain outcomes at mild and severe levels. *b* indicates the standardized beta coefficient. *x* indicates the values of prior-phase alcohol outcomes. *F* is the standard normal function. More details can also be found in the user guide of Mplus 8.0.

**Calculation of 95% uncertainty interval for marginal effect**

Since the conversion from regression coefficients to marginal effects in ordinal probit models involves non-linear transformations through the standard normal cumulative distribution function, directly transforming the 95% confidence intervals of beta coefficients does not yield valid uncertainty intervals for the marginal effects. To properly quantify uncertainty, we employed Monte Carlo simulation with 1,000 draws from the sampling distribution of the beta coefficients (assuming normality based on asymptotic theory). For each draw, we calculated the marginal effects as the change in predicted probabilities for each outcome category given a one standard deviation increase in latent response variables of alcohol consumption or pain severity levels, evaluated at the sample mean. The 2.5th and 97.5th percentiles of the simulated marginal effects distribution provided the 95% uncertainty intervals, which appropriately account for the non-linear transformation and ensure valid statistical inference. All analyses were conducted through R 4.0.2 software.

**Mplus code for random-intercept cross-lagged panel model (RI-CLPM)**

**Title:** RI-CLPM

Association between pure alcohol units and overall pain severity

**Data**: File is “…”;

**Variable**:

Names are …; ! All variables names

Usevariables are palcu1 palcu2 palcu3 pain1 pain2 pain3

…; ! Alcohol, pain, and other covariates names used in this analysis

Categorical are pain1 pain2 pain3; ! Added categorical/ordinal variables of the study outcomes

idvariable = …;

missing = palcu1-paclu3 pain1-pain3 (999);

**Analysis**:

Estimator = Bayes;

BITERATION = (8000);

Thin = 10;

**Model**:

ix BY palcu1-palcu3@1;

palcu2^-palcu3 PON palcu1^-palcu2; ! ^ indicates the within-person deviations

iy BY pain1-pain3@1;

pain2^-pain3^ PON pain1^-pain2^ ;

iy ix on …; ! iy and ix are latent variables

palcu2^-palcu3^ PON pain1^-pain2^;

pain2^-pain3^ PON palcu1^-palcu2^ ;

palcu1^-palcu3^ PWITH pain1^-pain3^;

**Output**: STDYX Residual Tech8 Tech10; ! STDYX offers estimations after standardization

**Plot**: Type = Plot3;

This script showed how we calculated the autoregressive and cross-lagged association between pure alcohol units and overall pain severity from adjusted RI-CLPM. Other analyses were similar to this script but with different datasets and variable names.

**Table S2**. Characteristics of weekly pure alcohol units, three pain metrics, baseline covariates, and retirement transitions status across three levels of overall pain severity and follow-up status (n = 6,679).

| **Variables** | **Overall pain severity in phase 7** | | | | **Loss to follow-up/Withdrawal in followed phase 9 and 12** | **Deaths in followed phases 9 and 12** |
| --- | --- | --- | --- | --- | --- | --- |
|  | **None** | **Mild pain** | **Moderate or severe pain** | **Missing** |  |  |
| N (%) | 1,160 (17.4) | 2,656 (39.8) | 1,103 (16.5) | 35 (0.5) | 1,045 (15.6) | 680 (10.2) |
| Pure alcohol units (median [IQR^a^]) | 10.0 [4.0, 18.0] | 9.0 [3.0, 18.0] | 7.00 [1.0, 16.0] | 10.0 [2.0, 24.0] | 6.0 [1.0, 15.0] | 7.0 [1.0, 16.0] |
| Level of alcohol consumption (%) |  |  |  |  |  |  |
| Non-drinkers | 37 (3.1) | 87 (3.3) | 85 (7.7) | 0 (0.0) | 94 (9.0) | 39 (5.7) |
| Infrequent drinkers | 187 (15.9) | 424 (15.9) | 248 (22.5) | 1 (16.7) | 230 (22.0) | 164 (24.1) |
| Low-to-moderate drinkers (>0–14 units/week) | 550 (46.7) | 1,274 (47.8) | 460 (41.7) | 2 (33.3) | 431 (41.2) | 278 (40.9) |
| Above-moderate drinkers (>14 units/week) | 385 (32.7) | 871 (32.6) | 296 (26.8) | 3 (50.0) | 260 (24.9) | 189 (27.8) |
| Missing | 18 (1.5) | 12 (0.4) | 14 (1.3) | 0 (0.0) | 30 (2.9) | 10 (1.5) |
| Indicator of alcohol dependency (%) |  |  |  |  |  |  |
| No | 952 (82.1) | 2,106 (79.3) | 858 (77.8) | 27 (77.1) | 861 (82.4) | 570 (83.8) |
| Yes | 208 (17.0) | 550 (20.7) | 243 (22.0) | 7 (20.0) | 176 (16.8) | 102 (15.0) |
| Missing | 0 (0.0) | 0 (0.0) | 2 (0.2) | 1 (2.9) | 8 (0.8) | 8 (1.2) |
| Sex: Women (%) | 222 (19.1) | 678 (25.5) | 453 (41.1) | 12 (34.3) | 383 (36.7) | 212 (31.2) |
| Age (mean [SD^b^]) | 60.1 [5.6] | 60.4 [5.8] | 60.6 [5.8] | 62.0 [5.4] | 62.4 [6.1] | 65.2 [5.9] |
| Socio-economic position (%) |  |  |  |  |  |  |
| High | 626 (54.0) | 1,360 (51.2) | 428 (38.8) | 11 (31.4) | 337 (32.2) | 282 (41.5) |
| Middle | 461 (39.7) | 1,112 (41.9) | 505 (45.8) | 17 (48.6) | 488 (46.7) | 302 (44.4) |
| Low | 69 (5.9) | 176 (6.6) | 166 (15.0) | 7 (20.0) | 215 (20.6) | 89 (13.1) |
| Missing | 4 (0.3) | 8 (0.3) | 4 (0.4) | 0 (0.0) | 5 (0.5) | 7 (1.0) |
| Employment transition (%) |  |  |  |  |  |  |
| Remained retired | 403 (34.7) | 1,070 (40.3) | 447 (40.5) | 12 (34.3) | 379 (36.3) | 310 (45.6) |
| Remained employed | 125 (10.8) | 242 (9.1) | 95 (8.6) | 7 (20.0) | 128 (12.2) | 62 (9.1) |
| Retirement transition | 570 (49.1) | 1,195 (45.0) | 445 (40.3) | 12 (34.3) | 156 (14.9) | 58 (8.5) |
| Other transitions | 62 (5.3) | 149 (5.6) | 116 (10.5) | 4 (11.4) | 59 (5.6) | 37 (5.4) |
| Missing | 0 (0.0) | 0 (0.0) | 0 (0.0) | 0 (0.0) | 323 (30.9) | 213 (31.3) |
| Retirement transition by age (%)^c^ |  |  |  |  |  |  |
| Midlife retirement transition | 232 (20.0) | 500 (18.8) | 202 (18.3) | 4 (11.4) | 83 (11.5) | 28 (6.0) |
| Retirement transition in later life | 338 (29.1) | 695 (26.2) | 243 (22.0) | 8 (22.9) | 73 (10.1) | 30 (6.4) |
| Smoking (%) |  |  |  |  |  |  |
| Non-smokers | 635 (54.7) | 1,405 (52.9) | 538 (48.8) | 8 (22.9) | 493 (47.2) | 295 (43.4) |
| Former smokers | 432 (37.2) | 1,026 (38.6) | 429 (38.9) | 8 (22.9) | 390 (37.3) | 283 (41.6) |
| Current smokers | 68 (5.9) | 175 (6.6) | 93 (8.4) | 1 (2.9) | 116 (11.1) | 87 (12.8) |
| Missing | 25 (2.2) | 50 (1.9) | 43 (3.9) | 18 (51.4) | 46 (4.4) | 15 (2.2) |
| BMI^d^ (%) |  |  |  |  |  |  |
| Without obesity (<30) | 960 (82.8) | 2,166 (81.6) | 795 (72.1) | 26 (74.3) | 669 (64.0) | 463 (68.1) |
| With obesity (≥30) | 156 (13.4) | 387 (14.6) | 263 (23.8) | 8 (22.9) | 216 (20.7) | 149 (21.9) |
| Missing | 44 (3.8) | 103 (3.9) | 45 (4.1) | 1 (2.9) | 160 (15.3) | 68 (10.0) |
| Jenkins sleep disturbance scores (median [IQR]) | 9.0 [6.0, 12.0] | 11.0 [8.0, 15.0] | 14.0 [10.0, 20.0] | 11.0 [7.0, 19.5] | 10.0 [7.0, 15.0] | 11.0 [8.0, 16.0] |
| Leisure-time physical activities (%) |  |  |  |  |  |  |
| Lower than recommended hours | 723 (62.3) | 1,719 (64.7) | 789 (71.5) | 10 (28.6) | 739 (70.7) | 467 (68.7) |
| Recommended hours or higher | 417 (35.9) | 910 (34.3) | 286 (25.9) | 7 (20.0) | 245 (23.4) | 183 (26.9) |
| Missing | 20 (1.7) | 27 (1.0) | 28 (2.5) | 18 (51.4) | 61 (5.8) | 30 (4.4) |
| RAND-36 Mental well-being score (median [IQR]) | 55.7 [51.3, 58.3] | 55.0 [49.4, 58.0] | 52.9 [44.2, 57.7] | 54.0 [50.5, 58.9] | 55.2 [48.8, 58.4] | 55.6 [48.8, 58.8] |
| Hypertension (%) | 393 (33.9) | 881 (33.2) | 457 (41.4) | 14 (40.0) | 497 (47.6) | 383 (56.3) |
| Cardiovascular disease (%) | 130 (11.2) | 404 (15.2) | 279 (25.3) | 8 (22.9) | 209 (20.0) | 206 (30.3) |
| Stroke (%) | 8 (0.7) | 18 (0.7) | 15 (1.4) | 0 (0.0) | 19 (1.8) | 22 (3.2) |
| Diabetes (%) | 25 (2.2) | 63 (2.3) | 31 (2.8) | 3 (8.6) | 58 (5.6) | 47 (6.9) |
| Respiratory illness (%) | 105 (9.1) | 331 (12.5) | 208 (18.9) | 3 (8.6) | 176 (16.8) | 131 (19.3) |
| Cancer (%) |  |  |  |  |  |  |
| No | 1013 (87.3) | 2,297 (86.5) | 971 (88.0) | 30 (85.7) | 901 (86.2) | 360 (52.9) |
| Yes | 147 (12.7) | 355 (13.4) | 132 (12.0) | 5 (14.3) | 143 (13.7) | 320 (47.1) |
| Missing | 0 (0.0) | 4 (0.2) | 0 (0.0) | 0 (0.0) | 1 (0.1) | 0 (0.0) |
| Prescribed analgesic use (%) | 109 (9.4) | 424 (16.0) | 419 (38.0) | 12 (34.3) | 104 (10.0) | 97 (14.3) |
| RAND-36 pain intensity score (%) |  |  |  |  |  |  |
| No pain | 1,144 (98.6) | 181 (6.8) | 3 (0.3) | 0 (0.0) | 270 (25.8) | 187 (27.5) |
| Mild pain | 0 (0.0) | 2,475 (93.2) | 144 (13.1) | 13 (37.1) | 490 (46.9) | 309 (45.4) |
| Moderate or severe pain | 0 (0.0) | 0 (0.0) | 953 (86.4) | 0 (0.0) | 266 (25.5) | 173 (25.4) |
| Missing | 16 (1.4) | 0 (0.0) | 3 (0.3) | 22 (62.9) | 19 (1.8) | 11 (1.6) |
| RAND-36 pain interference score (%) |  |  |  |  |  |  |
| No pain interference | 1143 (98.5) | 1,931 (72.7) | 198 (18.0) | 12 (34.3) | 597 (57.1) | 387 (56.9) |
| Mild pain interference | 0 (0.0) | 725 (27.3) | 404 (36.6) | 1 (2.9) | 253 (24.2) | 164 (24.1) |
| Moderate or severe pain interference | 0 (0.0) | 0 (0.0) | 498 (45.1) | 0 (0.0) | 173 (16.6) | 117 (17.2) |
| Missing | 17 (1.5) | 0 (0.0) | 3 (0.3) | 22 (62.9) | 22 (2.1) | 12 (1.8) |
| Number of recent MSP^e^ sites (%) |  |  |  |  |  |  |
| No pain (0 sites) | 1154 (97.4) | 1388 (52.3) | 277 (25.1) | 1 (2.9) | 596 (57.0) | 383 (56.3) |
| Localized pain (1–2 sites) | 0 (0.0) | 1268 (47.7) | 479 (43.4) | 5 (14.3) | 315 (30.1) | 237 (34.9) |
| Multisite pain (>2 sites) | 0 (0.0) | 0 (0.0) | 340 (30.8) | 1 (2.9) | 107 (10.2) | 44 (6.5) |
| Missing | 6 (0.5) | 0 (0.0) | 7 (0.6) | 28 (80.0) | 27 (2.6) | 16 (2.4) |

^a^ IQR: Interquartile range

^b^ SD: Standard deviation

^c^ It only represent the subgroups by age among participants experiencing retirement transition.

^d^ BMI: Body mass index

^e^ MSP: Musculoskeletal pain

**Table S3**. Missingness of baseline characteristics.

| **Variables** | **Missingness (N, %)** |
| --- | --- |
| Pure alcohol units | 75 (1.1) |
| Overall pain severity | 54 (0.8) |
| Sex | 0 (0.0) |
| Age | 0 (0.0) |
| Socioeconomic position (SEP) | 28 (0.4) |
| Current employment status | 0 (0.0) |
| Cigarette smoking status | 197 (2.8) |
| Body mass index (BMI) | 421 (6.0) |
| Sleep quality | 144 (2.1) |
| Leisure-time physical activities | 184 (2.6) |
| Hypertension | 0 (0.0) |
| Cardiovascular diseases | 0 (0.0) |
| Stroke | 0 (0.0) |
| Diabetes | 0 (0.0) |
| History of respiratory illness | 0 (0.0) |
| Cancer | 5 (0.0) |
| RAND-36 mental component score | 227 (3.3) |
| Prescribe analgesic medication | 0 (0.0) |
| Total missingness | 550 (7.9) |

**Table S4**. Loss to follow-up and deaths by phases.

| **Follow-up status** | **Phase 7** | **Phase 9** | **Phase 12** |
| --- | --- | --- | --- |
| Participated (N, %) | 6,679^a^ (95.9) | 6,060 (87.0) | 5,038 (72.3) |
| Loss to follow-up (N, %) | 0 (0.0) | 406 (5.8) | 890 (12.8) |
| Deaths (N, %) | 0 (0.0) | 213 (3.1) | 751 (10.8) |
| Missingness in time-variant alcohol and pain outcomes^b^ | | | |
| Pure alcohol units (N, %) | 75 (1.1) | 47 (0.7) | 70 (1.0) |
| Overall pain severity (N, %) | 67 (1.0) | 58^c^ (0.8) | 53 (0.8) |

^a^ 288 participants (4.1%) were excluded because they did not complete crucial questionnaires in phases 7, 9, and 12.

^b^ Loss to follow-up or deaths were not included.

^c^ Some individuals lost to follow-up in phase 9 and died in phase 12.

**Table S5**. Pooled associations between alcohol consumption and overall pain severity from fully-adjusted RI-CLPM with equality constraints on autoregressive and cross-lagged pathways, stratified by age at transition (midlife vs. older adults) among participants experiencing retirement transition.

|  | **Subsequent alcohol outcomes^a^** | **Subsequent pain outcomes^b^** |
| --- | --- | --- |
| **Among midlife participants experiencing retirement transition (n = 1,021)** | **Standardized beta (95% CI)** | **Standardized beta (95% CI)** |
| Pure alcohol units (Phase 7) | 0.16 (0.03, 0.29) | 0.06 (-0.05, 0.18) |
| Pure alcohol units (Phase 9) | -0.18 (-0.46, 0.05) | 0.15 (0.04, 0.25) |
| Overall pain severity (Phase 7) | 0.06 (-0.05, 0.16) | 0.04 (-0.12, 0.22) |
| Overall pain severity (Phase 9) | -0.04 (-0.17, 0.09) | 0.17 (0.02, 0.32) |
|  |  |  |
| **Among older participants experiencing retirement transition (n = 1,357)** |  |  |
| Pure alcohol units (Phase 7) | 0.26 (0.15, 0.36) | 0.08 (-0.01, 0.18) |
| Pure alcohol units (Phase 9) | -0.05 (-0.28, 0.14) | -0.07 (-0.16, 0.03) |
| Overall pain severity (Phase 7) | -0.02 (-0.11, 0.08) | 0.13 (-0.01, 0.27) |
| Overall pain severity (Phase 9) | 0.11 (-0.01, 0.22) | 0.01 (-0.14, 0.16) |

The Bayesian PPC p-value for analyses among midlife and older participants experiencing retirement transition are 0.282 and 0.311, respectively.

^a^ Subsequent alcohol outcome: within-person autoregressive associations between alcohol at phase 7 and alcohol at phase 9, and association between alcohol at phase 9 and alcohol at phase12; within-person cross-lagged association between pain at phase 7 and alcohol at phase 9, and association between pain at phase 9 and alcohol at phase 12.

^b^ Subsequent pain outcome: within-person autoregressive associations between pain at phase 7 and pain at phase 9, and association between pain at phase 9 and pain at phase12; within-person cross-lagged association between alcohol at phase 7 and pain at phase 9, and association between alcohol at phase 9 and pain at phase 12.

**Table S6.** Pooled associations between alcohol consumption and overall pain severity from fully-adjusted RI-CLPM without equality constraints on autoregressive and cross-lagged pathways, stratified by socioeconomic position (SEP) (high, intermediate, and low).

|  | **Subsequent alcohol outcomes^a^** | **Subsequent pain outcomes^b^** |
| --- | --- | --- |
| **Prior-phase variables** | **Standardized beta (95% CI)** | **Standardized beta (95% CI)** |
| **Among low-SEP participants (n = 614)** |  |  |
| Pure alcohol units (Phase 7) | 0.30 (0.19, 0.40) | 0.05 (-0.15, 0.24) |
| Pure alcohol units (Phase 9) | 0.17 (-0.08, 0.39) | 0.22 (0.04, 0.51) |
| Overall pain severity (Phase 7) | 0.06 (-0.05, 0.18) | -0.07 (-0.30, 0.21) |
| Overall pain severity (Phase 9) | 0.13 (-0.27, 0.49) | 0.06 (-0.16, 0.29) |
|  |  |  |
| **Among intermediate-SEP participants (n = 2,555)** | | |
| Pure alcohol units (Phase 7) | 0.24 (0.16, 0.31) | 0.06 (-0.02, 0.14) |
| Pure alcohol units (Phase 9) | -0.42 (-0.73, -0.20) | 0.00 (-0.07, 0.06) |
| Overall pain severity (Phase 7) | 0.05 (-0.02, 0.11) | 0.08 (-0.02, 0.19) |
| Overall pain severity (Phase 9) | 0.05 (-0.08, 0.17) | 0.10 (0.00, 0.21) |
|  |  |  |
| **Among high-SEP participants (n = 2,739)** | | |
| Pure alcohol units (Phase 7) | 0.38 (0.31, 0.44) | 0.06 (0.00, 0.13) |
| Pure alcohol units (Phase 9) | -0.18 (0.51, 0.04) | 0.03 (-0.04, 0.10) |
| Overall pain severity (Phase 7) | 0.04 (-0.02, 0.10) | 0.12 (0.02, 0.22) |
| Overall pain severity (Phase 9) | -0.03 (-0.13, 0.08) | 0.09 (-0.01, 0.19) |

The Bayesian PPC p-value for analyses among low-, intermediate-, and high-SEP participants are 0.364, 0.397, and 0.316.

^a^ Subsequent alcohol outcome: within-person autoregressive associations between alcohol at phase 7 and alcohol at phase 9, and association between alcohol at phase 9 and alcohol at phase12; within-person cross-lagged association between pain at phase 7 and alcohol at phase 9, and association between pain at phase 9 and alcohol at phase 12.

^b^ Subsequent pain outcome: within-person autoregressive associations between pain at phase 7 and pain at phase 9, and association between pain at phase 9 and pain at phase12; within-person cross-lagged association between alcohol at phase 7 and pain at phase 9, and association between alcohol at phase 9 and pain at phase 12.

**Table S7**. Association between alcohol consumption and overall pain severity from fully-adjusted RI-CLPM through completed case analysis.

|  | **Subsequent alcohol consumption** | **Subsequent overall pain severity** |
| --- | --- | --- |
| **Prior-phase variables** | **Standardized beta (95% CI)** | **Standardized beta (95% CI)** |
| **Among all participants (n = 4,249)** |  |  |
| Pure alcohol units (Phase 7) | 0.33 (0.28, 0.38) | 0.06 (0.01, 0.12) |
| Pure alcohol units (Phase 9) | -0.16 (-0.32, -0.02) | 0.01 (-0.04, 0.06) |
| Overall pain severity (Phase 7) | 0.02 (-0.03, 0.06) | 0.12 (0.04, 0.20) |
| Overall pain severity (Phase 9) | 0.04 (-0.04, 0.11) | 0.10 (0.02, 0.18) |

The Bayesian PPC p-value is 0.367.

**Table S8.** Correlation between baseline alcohol consumption as well as pain metrics and follow-up status at phases 9 and 12.

| **Variables** | **Participated** | **Non-response/Withdrawals** | **Died** | **p-values^a^** |
| --- | --- | --- | --- | --- |
| Pure alcohol units (median, [IQR]) | 9.0 [3.0, 18.0] | 6.0 [1.0, 15.0] | 7.0 [1.0, 16.0] | <0.001 |
| Indicator of alcohol dependency (%) | 492 (10.1) | 94 (9.4) | 55 (8.4) | 0.325 |
| RAND-36 bodily pain intensity |  |  |  | <0.001 |
| None | 1,328 (27.0) | 270 (26.3) | 187 (28.0) |  |
| Mild | 2,632 (53.6) | 490 (47.8) | 309 (46.2) |  |
| Moderate or severe | 953 (19.4) | 266 (25.9) | 173 (25.9) |  |
| RAND-36 bodily pain interference |  |  |  | <0.001 |
| None | 3,284 (66.9) | 597 (58.4) | 387 (57.9) |  |
| Mild | 1,130 (23.0) | 253 (24.7) | 164 (24.6) |  |
| Moderate or higher | 498 (10.1) | 173 (16.9) | 117 (17.5) |  |
| Number of MSP sites |  |  |  | 0.021 |
| None | 2,796 (56.9) | 596 (58.5) | 383 (57.7) |  |
| Localized | 1,698 (34.6) | 315 (30.9) | 237 (35.7) |  |
| Multisite | 419 (8.5) | 107 (10.5) | 44 (6.6) |  |
| Overall pain severity |  |  |  | <0.001 |
| None | 1,113 (22.7) | 225 (22.1) | 161 (24.3) |  |
| Mild/localized | 2,601 (53.1) | 479 (47.0) | 303 (35.6) |  |
| Moderate or severe/multisite | 1,181 (24.1) | 316 (31.0) | 200 (30.2) |  |

^a^ We use the Wilcox test for non-normally distributed pure alcohol units and Pearson chi-square test for ordinal pain outcomes.

**Sensitivity analyses under different missingness assumptions (MAR vs. NMAR)**

We conducted pattern-mixture sensitivity analyses to evaluate if MAR (FIML for loss to follow-up) and MCAR assumptions (deletion of deaths) are held. Since we hardly find any practical Mplus examples applying pattern mixture model or selection model to RI-CLPM, we implemented an alternative approach, the linear growth curve model (LGM), to estimate the potential effect of different pain severity levels on alcohol trajectories and the potential effect of alcohol consumption on pain trajectories under different missingness assumptions (Staudt et al. 2022). If MAR assumption were held in our pattern-mixture analyses using LGM, the missingness from loss to follow-up and deaths would theoretically be at random as we conducted RI-CLPM.

All missing patterns of loss to follow-up and deaths were summarized below:

**Table S9**. Missingness patterns of loss to follow-up and deaths in phases 9 and 12.

| **Pattern #** | **Phase 7** | **Phase 9** | **Phase 12** | **N (%)** |
| --- | --- | --- | --- | --- |
| 1 | Participated | Participated | Participated | 4,954 (71.1) |
| 2 | Participated | Participated | Dead | 467 (6.7) |
| 3 | Participated | Participated | Non-response/withdrawal | 639 (9.2) |
| 4 | Participated | Non-response/withdrawal | Participated | 84 (1.2) |
| 5 | Participated | Dead | Dead | 213 (3.1) |
| 6 | Participated | Non-response/withdrawal | Dead | 71 (1.0) |
| 7 | Participated | Non-response/withdrawal | Non-response/withdrawal | 251 (3.6) |

There are six different patterns of missingness from loss to follow-up and deaths. We assigned participants who participated in the study in three phases as the “all data” group (Pattern #1).

We assigned those who partially participated in two phases as the “partial data” group (Patterns #2, #3).

The remaining participants who only have the baseline information were assigned in the “no follow-up” group (Patterns #4, #5, #6, and #7).

In the “all data” group, there are 731 (10.5%) participants with missingness among time-variant alcohol and pain as well as baseline covariates.

Our sensitivity analyses were conducted in three steps: the first step was to perform completed case analyses among participants with completed information (n = 4,223); the second step was to apply MICE with random forest algorithms to handle the missingness in covariates and full-information maximum likelihood (FIML) estimate to LGM among participants who were alive across three phases (n = 5,928) assuming missingness is at random; the third step is to apply pattern-mixture analyses to LGM among all participants (n = 6,679).

Given that only three measurement occasions were available (Phases 7, 9, and 12), we specified linear growth models (intercept and slope) because the quadratic term typically requires at least four time points to acquire an adequate fit. To reflect potential unequal intervals among three phases (phase 7 → 9: 5 years; phase 9 → 12: 8 years, on average), factor loading for three alcohol indictors were set as 0 (phase 7), 1 (phase 9), and 2.6 (phase 12), respectively, assuming one year is equivalent to 0.2. The value of Bayesian informative criteria (BIC) was used for assessing model performance.

The intercept and slope were regressed on dummy variables of overall pain severity at baseline (mild pain vs. none, moderate to severe pain vs. none), adjusted to other baseline covariates. In the pattern-mixture analysis using LGM, we applied three parameter restriction methods, including complete case restriction, neighbouring case restriction, and available case restriction, for groups with insufficient data to estimate slopes.

The complete case restriction fixed the inestimable slope parameter (e.g. “no follow-up” group) for dropouts to equal that of study completers (e.g. “all data group”), assuming dropouts would have changed similarly to those who completed all assessments. The neighbouring case restriction fixed the inestimable slope parameter for dropouts to equal that of the most similar group (e.g. “partial data” group), assuming dropouts is similar to those who also experienced intermittent missingness. The available case restriction fixed the inestimable slope parameter for dropouts to the weighted average of all other groups' estimates, representing a neutral assumption that dropouts would change like the average of all observed participants.

In the LGM against pure alcohol units, we calculated the difference in predicted alcohol units across none, mild, and moderate-to-severe pain levels at phase 9 and 12. The findings from step 3 were compared to findings from step 1 and step 2 to evaluate if assumption of MAR and MCAR were held.

**Table S10**. Group differences and difference in change of predicted pure alcohol units across three levels of overall pain severity at phase 9 and 12.

| **Phases** | **Overall severity pain levels** | **Completed case analyses (n = 4,223)** | **With FIML estimates (n = 5,928)** | **Pattern mixture model with complete case restriction (n = 6,679)** | **Pattern mixture model with neighbouring case restriction (n = 6,679)** | **Pattern mixture model with available case restriction (n = 6,679)** |
| --- | --- | --- | --- | --- | --- | --- |
| **Group difference** | | | | | | |
| 9 | Mild pain vs. none | 0.012 (95% CI^a^: -0.714, 0.737) | 0.262 (95% CI: -0.391, 0.915) | 0.232 (95% CI: -0.410, 0.873) | 0.234 (95% CI: -0.426, 0.894) | 0.233 (95% CI: -0.412, 0.877) |
| 9 | Severe pain vs. none | -0.728 (95% CI: -1.643, 0.188) | -0.172 (95% CI: -0.988, 0.644) | -0.273 (95% CI: -1.064, 0.518) | -0.248 (95% CI: -1.059, 0.563) | -0.268 (95% CI: -1.062, 0.526) |
| 12 | Mild pain vs. none | -0.312 (95% CI: -1.118, 0.494) | -0.039 (95% CI: -0.765, 0.686) | -0.031 (95% CI: -0.826, 0.765) | -0.026 (95% CI: -0.942, 0.890) | -0.028 (95% CI: -0.844, 0.789) |
| 12 | Severe pain vs. none | -1.005 (95% CI: -2.023, 0.012) | -0.550 (95% CI: -1.460, 0.360) | -0.579 (95% CI: -1.549, 0.392) | -0.512 (95% CI: -1.611, 0.587) | -0.564 (95% CI: -1.557, 0.428) |
| **Difference in change** | | | | | | |
| 9 | Mild pain vs. none | -0.203 (95% CI: -0.469, 0.064) | -0.188 (95% CI: -0.434, 0.058) | -0.164 (95% CI: -0.449, 0.122) | -0.163 (95% CI: -0.503, 0.178) | -0.163 (95% CI: -0.459, 0.133) |
| 9 | Severe pain vs. none | -0.174 (95% CI: -0.510, 0.163) | -0.236 (95% CI: -0.546, 0.073) | -0.191 (95% CI: -0.540, 0.157) | 0.165 (95% CI: -0.572, 0.242) | -0.185 (95% CI: -0.545, 0.174) |
| 12 | Mild pain vs. none | -0.527 (95% CI: -1.219, 0.166) | -0.489 (95% CI: -1.129, 0.150) | -0.426 (95% CI: -1.169, 0.317) | -0.423 (95% CI: -1.309, 0.463) | -0.423 (95% CI: -1.192, 0.346) |
| 12 | Severe pain vs. none | -0.451 (95% CI: -1.326, 0.423) | -0.614 (95% CI: -1.419, 0.190) | -0.497 (95% CI: -1.403, 0.409) | 0.429 (95% CI: -1.487, 0.629) | -0.482 (95% CI: -1.416, 0.452) |

Positive difference indicates that predicted pure alcohol units among individuals with mild/severe pain are greater than those among individuals without any pain at phase 7 or 9.

^a^ Null hypothesis is that difference in predicted pure alcohol units between two pain levels is 0. If the 95% CI does not include 0, we did not reject the null hypothesis.

In the LGM with ordinal overall pain severity, the categorical pain outcomes were converted into continuous latent response variables based on item-response theory and factor analytic frameworks. We applied the same factor loading (0, 1, 2.6) for pain indicators at three phases. The alcohol consumption at baseline was categorized into four levels: non-drinkers or infrequent drinkers (0 units), low-to-moderate drinkers (>0–14 units/week), increase-risk drinkers (women: >14–35 units/week; men: >14–50 units/week), and excessive drinkers (women: >35 units/week; men: >50 units/week) (NHS England 2025). The intercept and slope were regressed on dummy variables of alcohol consumption levels at baseline (non-drinkers/infrequent drinkers vs. low-to-moderate drinkers [>0-14 units/week], increase-risk drinkers [Women: >14–35 units/week; Men: >14–50 units/week] vs. low-to-moderate drinkers, and excessive drinkers [Women: >35 units/week; Men: >50 units/week] vs. low-to-moderate drinkers), adjusted to other baseline covariates.

Similarly, we applied three parameter restriction methods for groups with insufficient data to estimate slopes in the pattern mixture analyses. At last, we calculated the difference in predicted latent pain severity across non-drinker/infrequent drinkers, low-to-moderate drinkers, increase-risk drinkers, and excessive drinkers at phases 9 and 12. The findings from step 3 were compared to findings from step 1 and step 2 to evaluate if assumption of MAR and MCAR were held.

**Table S11**. Difference in change (from baseline) of predicted latent response variables of overall pain severity across four different alcohol consumption levels at phase 9 and 12.

| **Phases** | **Weekly pure alcohol consumption** | **Completed case analyses^a^ (n = 4,223)** | **With FIML estimates^b^ (n = 5,928)** | **Pattern mixture model^c^ with complete case restriction (n = 6,679)** | **Pattern mixture model with neighbouring case restriction (n = 6,679)** | **Pattern mixture model with available case restriction (n = 6,679)** |
| --- | --- | --- | --- | --- | --- | --- |
| 9 | Non-drinkers or infrequent drinkers (0 units) vs. low-to-moderate drinkers (>0-14 units) | -0.039 (95% CI^d^: -0.107, 0.015) | -0.079 (95% CI: -0.184, 0.026) | -0.032 (95% CI: -0.147, 0.084) | -0.005 (95% CI: -0.139, 0.129) | -0.026 (95% CI: -0.145, 0.093) |
| 9 | Increasing-risk drinkers (>14-35 units for women; >14-50 units for men) vs. low-to-moderate drinkers | 0.019 (95% CI: -0.029, 0.056) | 0.002 (95% CI: -0.073, 0.078) | -0.008 (95% CI: -0.096, 0.080) | -0.016 (95% CI: -0.123, 0.091) | -0.010 (95% CI: -0.101, 0.082) |
| 9 | Excessive drinkers (>35 units for women; >50 units for men) vs. low-to-moderate drinkers | 0.109 (95% CI: -0.057, 0.238) | 0.082 (95% CI: -0.169, 0.334) | 0.284 (95% CI: -0.007, 0.575) | 0.373 (95% CI: 0.024, 0.722) | 0.302 (95% CI: 0.001, 0.602) |
| 12 | Non-drinkers or infrequent drinkers (0 units) vs. low-to-moderate drinkers (>0-14 units) | -0.100 (95% CI: -0.277, 0.040) | -0.206 (95% CI: -0.479, 0.067) | -0.082 (95% CI: -0.383, 0.219) | -0.012 (95% CI: -0.361, 0.336) | -0.068 (95% CI: -0.377, 0.241) |
| 12 | Increase-risk drinkers (>14-35 units for women; >14-50 units for men) vs. low-to-moderate drinkers | 0.049 (95% CI: -0.075, 0.146) | 0.006 (95% CI: -0.190, 0.203) | -0.021 (95% CI: -0.250, 0.208) | -0.041 (95% CI: -0.319, 0.237) | -0.025 (95% CI: -0.263, 0.213) |
| 12 | Excessive drinkers (>35 units for women; >50 units for men) vs. low-to-moderate drinkers | 0.283 (95% CI: -0.149, 0.619) | 0.214 (95% CI: -0.439, 0.868) | 0.738 (95% CI: -0.018, 1.494) | 0.970 (95% CI: 0.061, 1.878) | 0.784 (95% CI: 0.002, 1.566) |

Group difference was not available in this case as latent response variable of overall pain severity was standardized at 0 at baseline.

Positive difference indicates that individuals with other levels of pure alcohol consumption may experience higher pain severity, compared to low-to-moderate alcohol consumption.

^a^ In the completed case analysis, threshold values for mild pain level at phase 9 and 12 are -0.512. Threshold values for severe pain level at phase 9 and 12 are 1.677.

^b^ Among alive participants, threshold values for mild pain level at phase 9 and 12 are 0.363. Threshold values for severe pain level at phase 9 and 12 are 4.108.

^c^ Among all participants, threshold values for mild pain level at phase 9 and 12 are -1.374. Threshold values for severe pain level at phase 9 and 12 are 2.334.

^d^ Null hypothesis is that difference of change is equal to 0. If the 95% CI does not include 0, we did not reject the null hypothesis.

**Reference**:

Staudt, Andreas, et al. (2022), 'Sensitivity analyses for data missing at random versus missing not at random using latent growth

modelling: a practical guide for randomised controlled trials', BMC Medical Research Methodology, 22 (1), 250.

NHS England (2015) 'Chapter 12: Alcohol',

<https://www.gov.uk/government/publications/delivering-better-oral-health-an-evidence-

based-toolkit-for-prevention/chapter-12-alcohol>, accessed 26 November.

**Table S12**. Pooled association between pure alcohol units and ordinal number of MSP sites through fully-adjusted RI-CLPM without equality constraints on autoregressive and cross-lagged panel pathways.

|  | **Subsequent alcohol outcomes** | **Subsequent pain outcomes** |
| --- | --- | --- |
| **Among all participants (n = 5928)** | **Standardized beta (95% CI)** | **Standardized beta (95% CI)** |
| Pure alcohol units (Phase 7) | 0.29 (0.25, 0.33) | 0.09 (0.03, 0.14) |
| Pure alcohol units (Phase 9) | -0.19 (-0.31, -0.08) | -0.01 (-0.06, 0.04) |
| Number of MSP sites (Phase 7) | -0.02 (-0.07, 0.02) | 0.18 (0.10, 0.26) |
| Number of MSP sites (Phase 9) | 0.08 (-0.01, 0.17) | 0.14 (0.06, 0.22) |

The Bayesian PPC p-value is 0.480.

**Table S13**. Pooled association between pure alcohol units and ordinal bodily pain intensity through fully-adjusted RI-CLPM without equality constraints on autoregressive and cross-lagged panel pathways.

|  | **Subsequent alcohol outcomes** | **Subsequent pain outcomes** |
| --- | --- | --- |
| **Among all participants (n = 5928)** | **Standardized beta (95% CI)** | **Standardized beta (95% CI)** |
| Pure alcohol units (Phase 7) | 0.28 (0.24, 0.32) | 0.06 (0.01, 0.11) |
| Pure alcohol units (Phase 9) | -0.20 (0.32, -0.10) | 0.03 (0.00, 0.08) |
| Bodily pain intensity (Phase 7) | 0.05 (0.01, 0.09) | 0.12 (0.05, 0.19) |
| Bodily pain intensity (Phase 9) | 0.04 (-0.04, 0.12) | 0.14 (0.07, 0.21) |

The Bayesian PPC p-value is 0.220.

**Table S14**. Pooled association between pure alcohol units and ordinal bodily pain interference through fully-adjusted RI-CLPM without equality constraints on autoregressive and cross-lagged panel pathways.

|  | **Subsequent alcohol outcomes** | **Subsequent pain outcomes** |
| --- | --- | --- |
| **Among all participants (n = 5928)** | **Standardized beta (95% CI)** | **Standardized beta (95% CI)** |
| Pure alcohol units (Phase 7) | 0.28 (0.24, 0.32) | 0.03 (-0.03, 0.08) |
| Pure alcohol units (Phase 9) | -0.20 (-0.32, -0.10) | 0.06 (0.02, 0.11) |
| Bodily pain interference (Phase 7) | 0.02 (-0.02, 0.06) | 0.16 (0.07, 0.25) |
| Bodily pain interference (Phase 9) | -0.04 (-0.13, 0.06) | 0.14 (0.05, 0.22) |

The Bayesian PPC p-value is 0.536.

**Table S15**. Loss to follow-up and deaths across phases, stratified by SEP at baseline.

| **SEP** | **High SEP: administrative** | **Intermediate SEP: Professional / executive** | **Low SEP: clerical / support** | **Missing^a^** |
| --- | --- | --- | --- | --- |
| Phase 7: participated (N) | 3,044 | 2,885 | 722 | 28 |
| Phase 9 |  |  |  |  |
| Participated (N, %) | 2,841 (93.3) | 2,588 (89.7) | 611 (84.6) | 20 (71.4) |
| Loss to follow-up (N, %) | 122 (4.0) | 195 (6.8) | 86 (11.9) | 3 (10.7) |
| Deaths^b^ (N, %) | 81 (2.7) | 102 (3.5) | 25 (3.5) | 5 (17.9) |
| Phase 12 |  |  |  |  |
| Participated (N, %) | 2,454 (80.6) | 2,137 (74.1) | 431 (59.7) | 16 (57.1) |
| Loss to follow-up (N, %) | 285 (9.4) | 418 (14.5) | 183 (25.3) | 4 (14.3) |
| Deaths (N, %) | 305 (10.0) | 330 (11.4) | 108 (15.0) | 8 (28.6) |

**Table S16**. Baseline characteristics against three levels of socioeconomic positions (SEP).

| **Variables** | **High SEP (administrative)** | **Intermediate SEP (Professional/executive)** | **Low SEP (clerical/support)** | **Missing** |
| --- | --- | --- | --- | --- |
| N | 3,044 | 2,885 | 722 | 28 |
| Sex: female (%) | 441 (14.5) | 972 (33.7) | 531 (73.5) | 16 (57.1) |
| Age (mean, SD^a^) | 61.0 (5.9) | 60.9 (6.0) | 63.1 (6.0) | 64.6 (6.0) |
| Weekly pure alcohol units (median, IQR^b^) | 12.0 [5.0, 20.0] | 7.0 [2.0, 15.0] | 2.0 [0.0, 6.0] | 2.0 [0.0, 7.0] |
| Levels of weekly alcohol consumption (%) |  |  |  |  |
| Non-drinkers | 70 (2.3) | 165 (5.7) | 101 (14.0) | 6 (21.4) |
| Infrequent drinkers | 301 (9.9) | 658 (22.8) | 287 (39.8) | 8 (28.6) |
| Low-to-moderate drinkers (>0–14 units) | 1,442 (47.4) | 1,304 (45.2) | 241 (33.4) | 8 (28.6) |
| Above-moderate drinkers (>14 units) | 1,207 (39.7) | 726 (25.2) | 67 (9.3) | 4 (14.3) |
| Missing | 24 (0.8) | 32 (1.1) | 26 (3.6) | 2 (7.1) |
| Indicator of alcohol dependency |  |  |  |  |
| No | 2,671 (87.7) | 2,529 (87.7) | 636 (88.1) | 25 (89.3) |
| Yes | 331 (10.9) | 284 (9.8) | 25 (3.5) | 1 (3.6) |
| Missing | 42 (1.4) | 72 (2.5) | 64 (8.4) | 2 (7.1) |
| Cigarette smoking status (%) |  |  |  |  |
| Non-smoker | 1,600 (52.6) | 1,379 (47.8) | 385 (53.3) | 10 (35.7) |
| Former smoker | 1,219 (40.0) | 1,136 (39.4) | 204 (28.3) | 9 (22.1) |
| Current smoker | 162 (5.3) | 287 (9.9) | 86 (11.9) | 5 (17.9) |
| Missing | 63 (2.1) | 83 (2.9) | 47 (6.5) | 4 (14.3) |
| BMI^c^ (%) |  |  |  |  |
| Without obesity (BMI<30) | 2,408 (79.1) | 2,199 (76.2) | 457 (63.3) | 15 (53.6) |
| With obesity (BMI≥30) | 449 (14.8) | 514 (17.8) | 209 (28.9) | 7 (25.0) |
| Missing | 187 (6.1) | 172 (6.0) | 56 (7.8) | 6 (21.4) |
| Leisure-time physical activities (%) |  |  |  |  |
| Lower than recommended level (<2.5 hours weekly) | 1,953 (64.2) | 1,958 (67.9) | 519 (71.9) | 17 (60.7) |
| At or above recommended level (≥2.5 hours weekly) | 1,053 (34.6) | 865 (30.0) | 125 (17.3) | 5 (17.9) |
| Missing | 38 (1.2) | 62 (2.1) | 78 (10.8) | 4 (14.3) |
| Sleep disturbance score (median, IQR) | 11.0 [8.0, 15.0] | 11.0 [8.0, 16.0] | 11.0 [8.0, 17.0] | 13.0 [6.3, 18.3] |
| Mental well-being score (median, IQR) | 55.2 [50.0, 58.1] | 54.8 [48.4, 58.3] | 54.0 [47.0, 58.1] | 54.4 [49.5, 57.6] |
| Hypertension: yes (%) | 1,104 (36.3) | 1,173 (40.7) | 332 (46.0) | 16 (57.1) |
| CVD^d^: yes (%) | 475 (15.6) | 579 (20.1) | 178 (24.7) | 4 (14.3) |
| Stroke: yes (%) | 26 (0.9) | 45 (1.6) | 9 (1.2) | 2 (7.1) |
| Diabetes yes (%) | 54 (1.8) | 118 (4.1) | 52 (7.2) | 2 (7.1) |
| History of respiratory illness: yes (%) | 389 (12.8) | 441 (15.3) | 118 (16.3) | 6 (21.4) |
| Cancer (%) |  |  |  |  |
| No | 2,496 (82.0) | 2,439 (84.5) | 613 (84.9) | 24 (85.7) |
| Yes | 547 (18.0) | 444 (15.4) | 107 (14.8) | 4 (14.3) |
| Missing | 1 (0.0) | 2 (0.1) | 2 (0.3) | 0 (0.0) |

^a^ SD: Standard deviation

^b^ IQR: Interquartile range (25^th^ percentile – 75^th^ percentile)

^c^ BMI: Body mass index

^d^ CVD: Cardiovascular diseases

**Table 17**. Baseline characteristics of alcohol consumption and pain severity stratified by MHS quartiles.

|  | **0–25^th^ percentile (MHS score: 0–68)** | **25–50^th^ percentile (MHS score: 68–80)** | **50–75^th^ percentile (MHS score: 80–88)** | **75–100^th^ percentile (MHS score: 88–100)** | **Missing** | **P-values^b^** |
| --- | --- | --- | --- | --- | --- | --- |
| N | 1,780 | 1,688 | 1,702 | 1,411 | 98 | - |
| **Pure alcohol units (median [IQR])** | 8.0 [2.0, 16.0] | 9.0 [3.0, 17.0] | 8.0 [3.0, 18.0] | 9.0 [3.0, 17.0] | 4.0 [0.0, 11.5] | <0.001 |
| **Levels of alcohol consumption (%)** |  |  |  |  |  |  |
| Non-drinkers | 117 (6.6) | 84 (5.0) | 70 (4.1) | 65 (4.6) | 6 (6.1) | <0.001 |
| Infrequent drinkers | 383 (21.5) | 296 (17.5) | 301 (17.7) | 260 (18.4) | 14 (14.3) |  |
| Low-to-moderate drinkers (>0–14 units/week) | 757 (42.5) | 770 (45.6) | 787 (46.2) | 651 (46.1) | 30 (30.6) |  |
| Above-moderate drinkers (>14 units/week) | 504 (28.3) | 528 (31.3) | 535 (31.4) | 425 (30.1) | 12 (12.2) |  |
| Missing | 19 (1.1) | 10 (0.6) | 9 (0.5) | 10 (0.7) | 36 (36.7) |  |
| **Indicator of alcohol dependency^a^ (%)** |  |  |  |  |  | <0.001 |
| No | 1,440 (80.9) | 1,490 (88.3) | 1,553 (91.2) | 1,325 (93.9) | 53 (54.1) |  |
| Yes | 283 (21.5) | 168 (10.0) | 122 (7.2) | 63 (4.5) | 5 (5.1) |  |
| Missing | 57 (3.2) | 30 (1.8) | 27 (1.6) | 23 (1.6) | 40 (40.8) |  |
| **Bodily pain intensity (%)** |  |  |  |  |  | <0.001 |
| None | 321 (18.0) | 405 (24.0) | 503 (29.6) | 543 (38.5) | 13 (13.3) |  |
| Mild pain | 885 (49.7) | 915 (54.2) | 925 (54.3) | 684 (48.5) | 22 (22.4) |  |
| Moderate or higher pain intensity | 571 (32.1) | 365 (21.6) | 271 (15.9) | 179 (12.7) | 6 (6.1) |  |
| Missing | 3 (0.2) | 3 (0.2) | 1 (0.1) | 5 (0.5) | 57 (58.2) |  |
| **Bodily pain interference (%)** |  |  |  |  |  | <0.001 |
| None | 844 (47.4) | 1,078 (63.9) | 1,227 (72.1) | 1,091 (77.3) | 28 (28.6) |  |
| Mild pain | 545 (30.6) | 415 (24.6) | 341 (20.0) | 236 (16.7) | 10 (10.2) |  |
| Moderate or higher pain interference | 385 (21.6) | 191 (11.3) | 131 (7.7) | 79 (5.6) | 2 (2.0) |  |
| Missing | 6 (0.3) | 4 (0.2) | 3 (0.2) | 5 (0.4) | 58 (59.2) |  |
| **Number of MSP sites (%)** |  |  |  |  |  | <0.001 |
| None | 851 (47.8) | 936 (55.5) | 1,043 (61.3) | 935 (66.3) | 57 (58.2) |  |
| Localized MSP (1-2 sites) | 669 (37.6) | 595 (35.2) | 574 (33.7) | 411 (29.1) | 36 (36.7) |  |
| Multisite MSP (>2 sites) | 260 (14.6) | 157 (9.3) | 85 (5.0) | 65 (4.6) | 5 (5.1) |  |
| **Overall pain severity (%)** |  |  |  |  |  | <0.001 |
| None | 264 (14.8) | 339 (20.1) | 424 (24.9) | 478 (33.9) | 24 (24.5) |  |
| Mild pain | 816 (45.8) | 909 (53.9) | 947 (55.6) | 716 (50.7) | 55 (56.1) |  |
| Moderate or higher pain severity | 700 (39.3) | 440 (26.1) | 331 (19.4) | 217 (15.4) | 19 (19.4) |  |

Lower MHS scores indicate poorer mental well-being.

^a^ We used ‘CAGE’ questionnaires to assess the potential alcohol dependency. It was not included in the primary analyses because the questionnaire was not available in Phase 12 and it measures lifetime alcohol dependency.

^b^ The Wilcoxon test (for continuous outcomes) and chi-squared test (for categorical outcomes) were implemented to evaluate the correlation between alcohol consumption/pain outcomes and MHS. P-values < 0.05 indicates that we fail to reject the null hypothesis that there is no correlation between MHS and alcohol/pain outcomes.

**Table S18**. Measurement invariance test for ordinal overall pain severity across three phases.

|  | **Without any constraints on threshold values on pain indicators** | **Adding constraints to threshold values on pain indicators** | **Apply approximal variance priors on threshold values (**$\boldsymbol{\sigma}^{\boldsymbol{2}}$ **= 0.1)** | **Apply approximal variance priors on threshold values (**$\boldsymbol{\sigma}^{\boldsymbol{2}}$ **= 0.01)** | **Apply approximal variance priors on threshold values (**$\boldsymbol{\sigma}^{\boldsymbol{2}}$ **= 0.001)** |
| --- | --- | --- | --- | --- | --- |
| **Intercepts for pure alcohol units** | | | | | |
| Phase 7 | 2.06 | 2.00 | 2.08 | 2.07 | 2.08 |
| Phase 9 | 1.89 | 1.87 | 1.95 | 1.95 | 1.95 |
| Phase 12 | 1.93 | 2.08 | 2.17 | 2.17 | 2.17 |
| **Threshold values for the level of mild or localized pain** | | | | | |
| Phase 7 | 0.379 | -0.757 | 0.195 | 0.116 | 0.213 |
| Phase 9 | 0.292 |  | 0.107 | 0.031 | 0.127 |
| Phase 12 | 0.205 |  | 0.022 | -0.050 | 0.077 |
| **Threshold values for the level of mild or localized pain** | | | | | |
| Phase 7 | 1.784 | 0.675 | 1.599 | 1.512 | 1.603 |
| Phase 9 | 1.758 |  | 1.573 | 1.483 | 1.541 |
| Phase 12 | 1.623 |  | 1.440 | 1.355 | 1.437 |
|  |  |  |  |  |  |
| **Model fit** | | | | | |
| PPC – mean difference of Pearson chi-square values | -318.9 | -675.1 | -310.8 | -400.8 | -1077.9 |
| PPC – p-values | 0.329 | 0.015 | 0.282 | 0.224 | 0.047 |
| Degree of freedom | 54 | 54 | 58 | 58 | 58 |

**Figure S1**. The framework of cross-lagged panel analysis (CLPM) in the study.


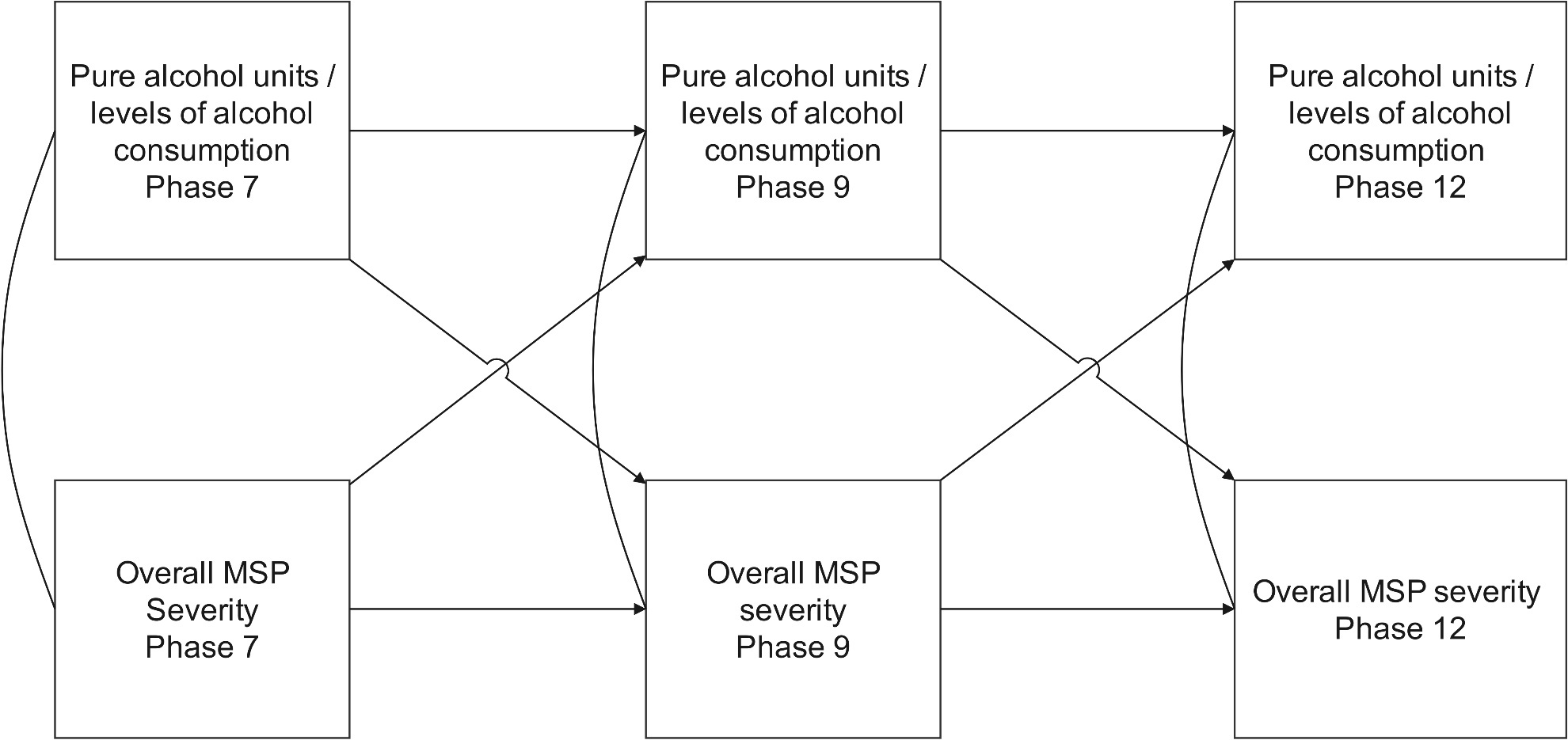


**Figure S2**. Spaghetti plots of within-person deviation of pure alcohol consumption over phases stratified by retirement transition status.

**
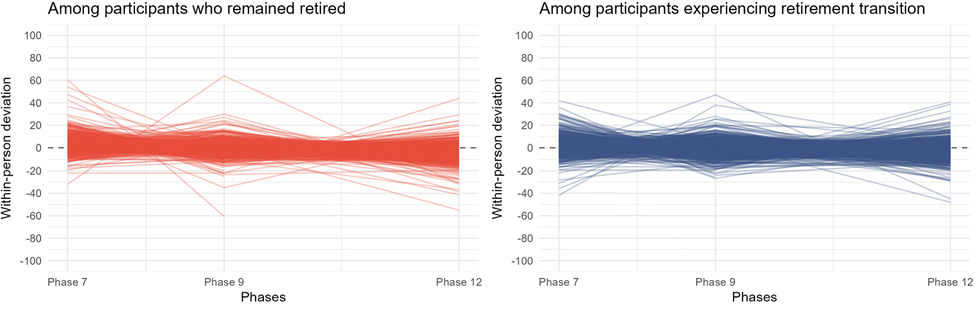
**

**
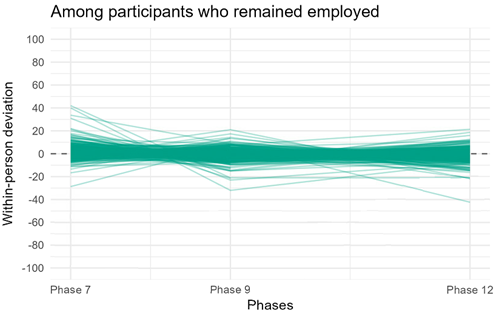
**

**Figure S3**. Spaghetti plots of within-person deviation of pure alcohol consumption over phases stratified by SEP strata.

**
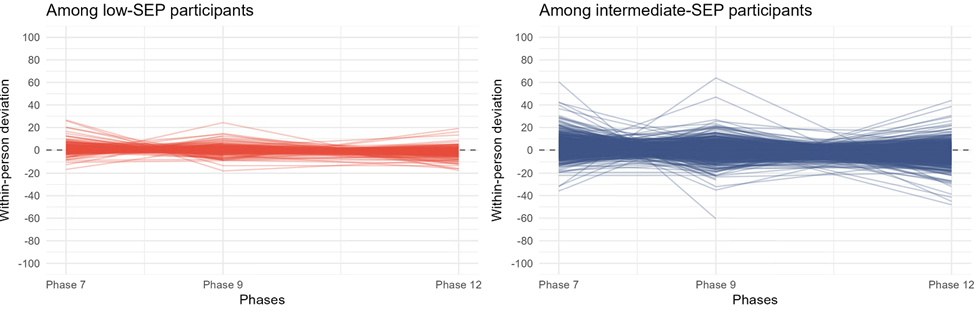
**

**
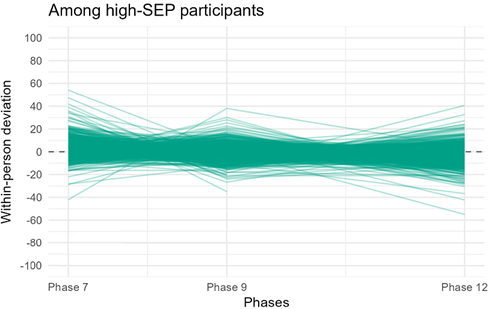
**

**Figure S4**. Spaghetti plots of within-person deviation of latent pain severity over phases stratified by retirement transition status.


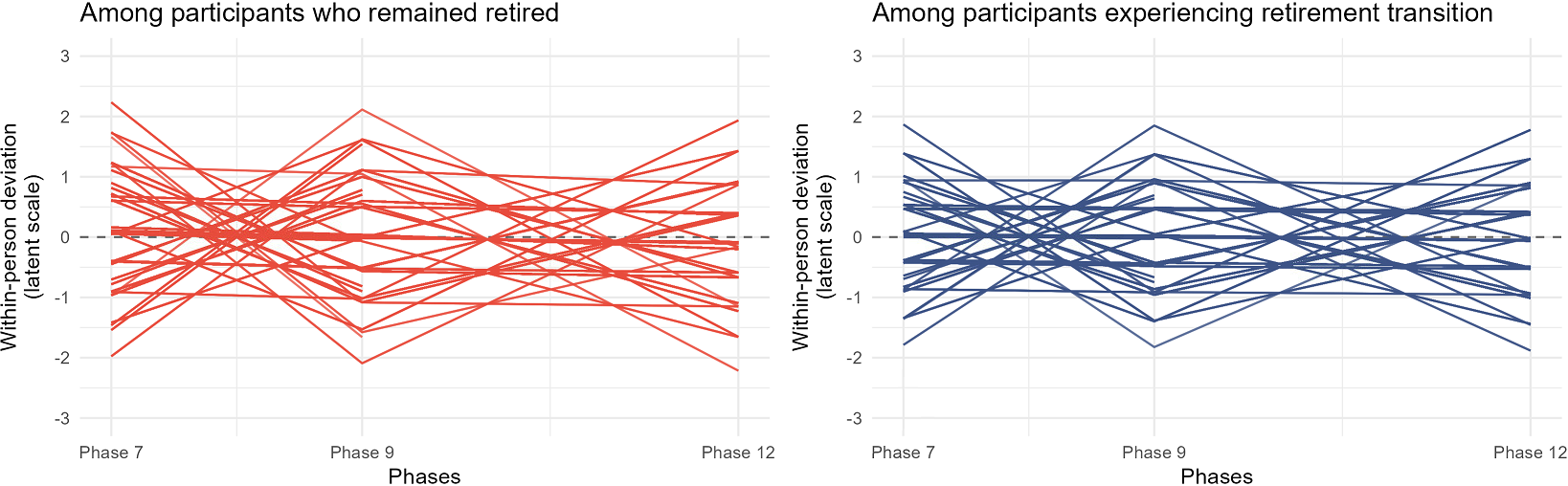


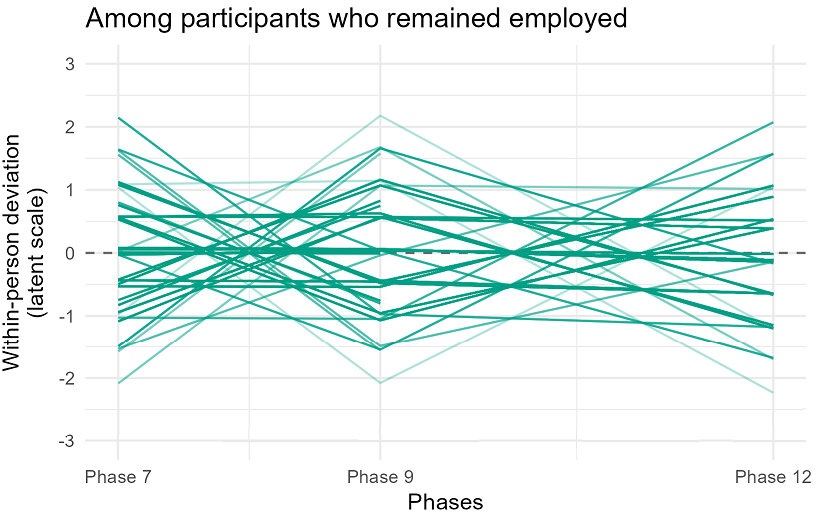


**Figure S5**. Spaghetti plots of within-person deviation of latent pain severity over phases stratified by SEP.


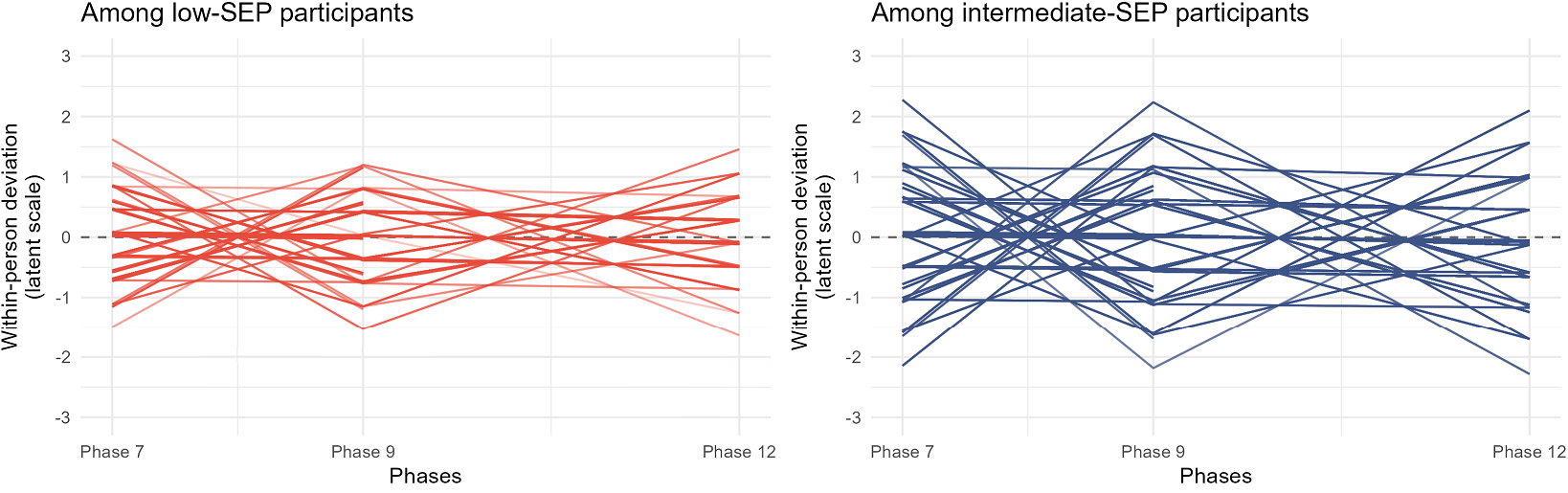


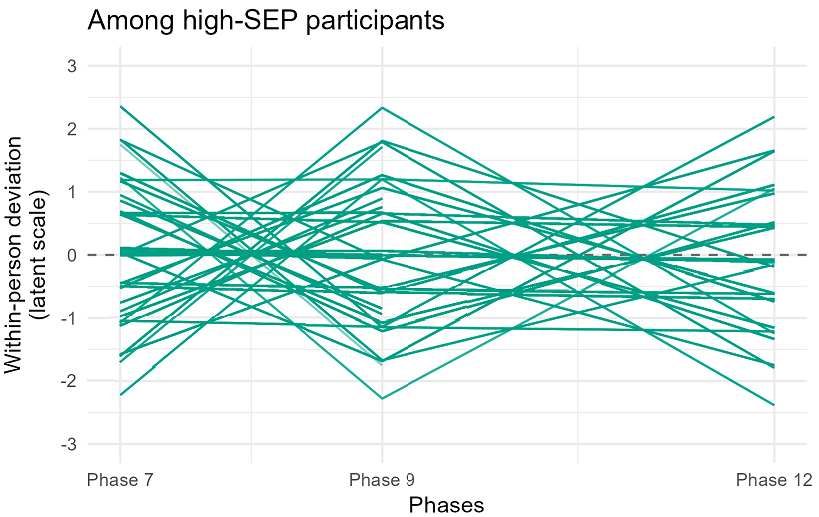


**Figure S6**. Marginal effect of probability increase of no-, mild-, and above-moderate-pain levels in subsequent phases per every standard deviation of pure alcohol consumption at phases 7 and 9.


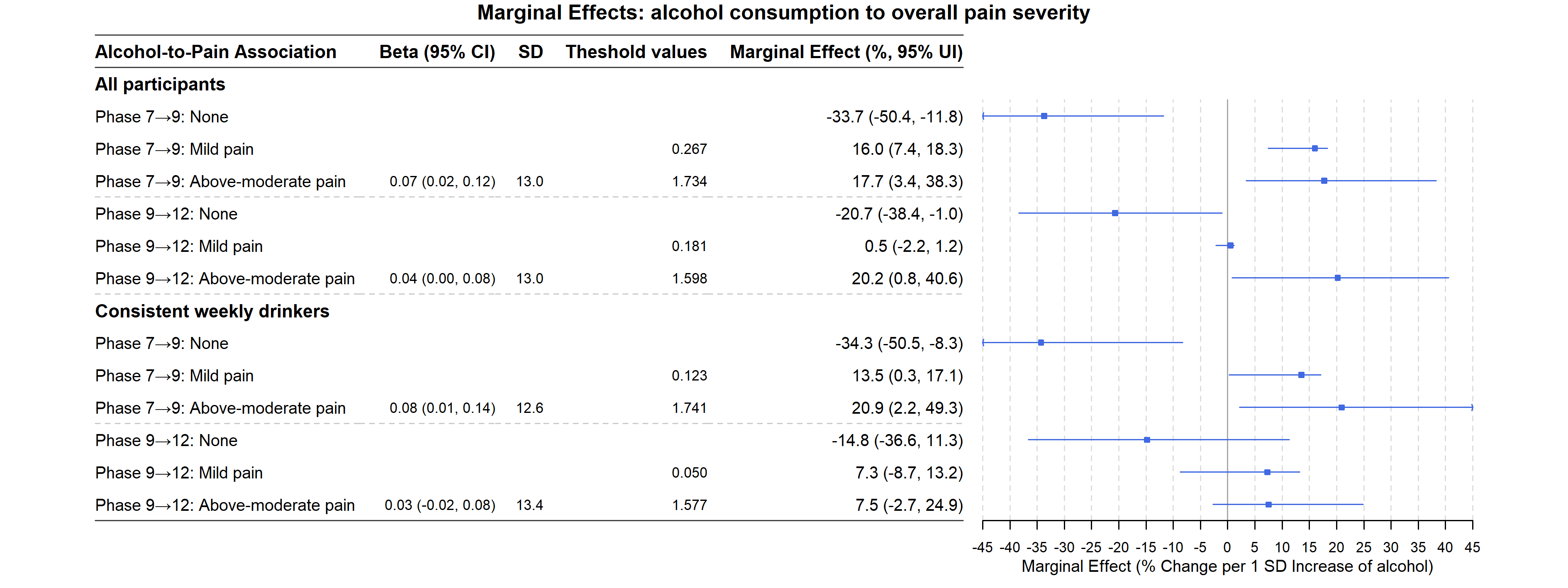


Different beta and 95% CI across three phases: we acquired the standardized beta from the fully-adjusted RI-CLPM without equality constraints.

SD: every standard deviation of pure alcohol units at two prior phases (phases 7 and 9).

Threshold values: threshold values for mild- and above-moderate-pain level at two subsequent phases (phases 9 and 12).

The 95% uncertainty intervals for marginal effects were computed through Monto-Carlo simulation with 1000 draws. Read “Calculation of 95% uncertainty interval for marginal effect” section for more details.

**Figure S7**. Marginal effect of probability increase of no-, mild-, and above-moderate-pain levels in subsequent phases per every standard deviation of pure alcohol consumption at phases 7 and 9 among participants who remained retired.


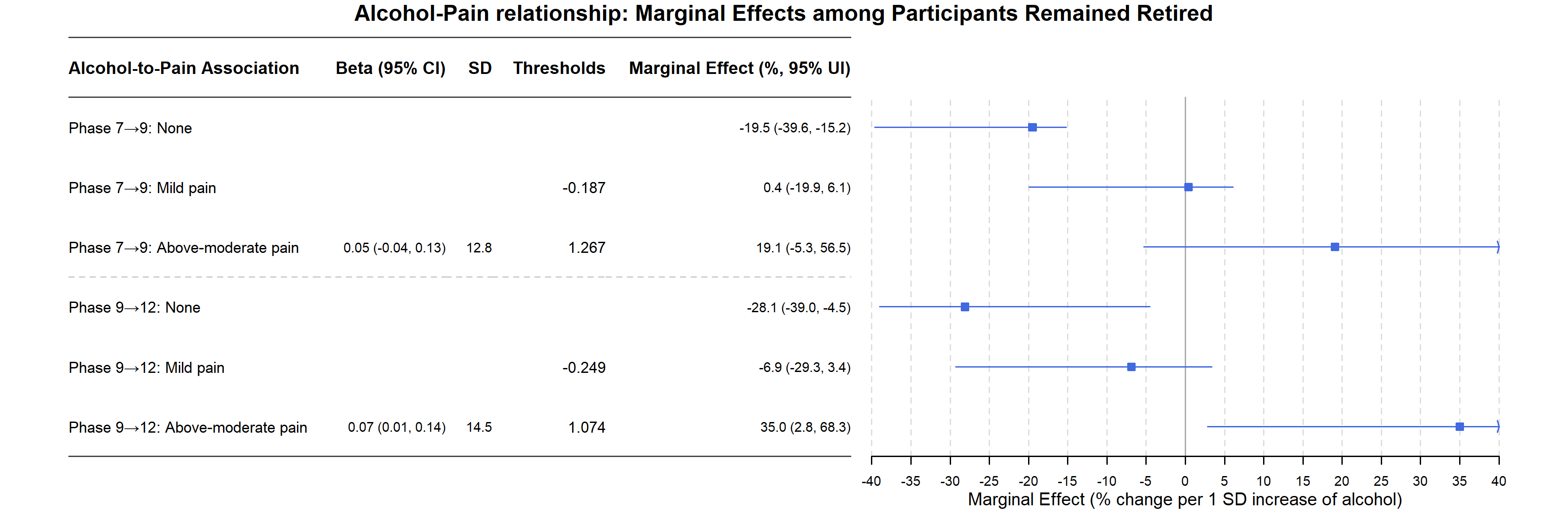


Different beta and 95% CI across three phases: we acquired the standardized beta from the fully-adjusted RI-CLPM without equality constraints.

SD: every standard deviation of pure alcohol units at two prior phases (phases 7 and 9).

Threshold values: threshold values for mild- and above-moderate-pain level at two subsequent phases (phases 9 and 12).

The 95% uncertainty intervals for marginal effects were computed through Monto-Carlo simulation with 1000 draws.

**Figure S8**. Marginal effect of alcohol consumption increases in subsequent phases per every standard deviation of latent pain severity at phases 7 and 9 among participants remained retired.


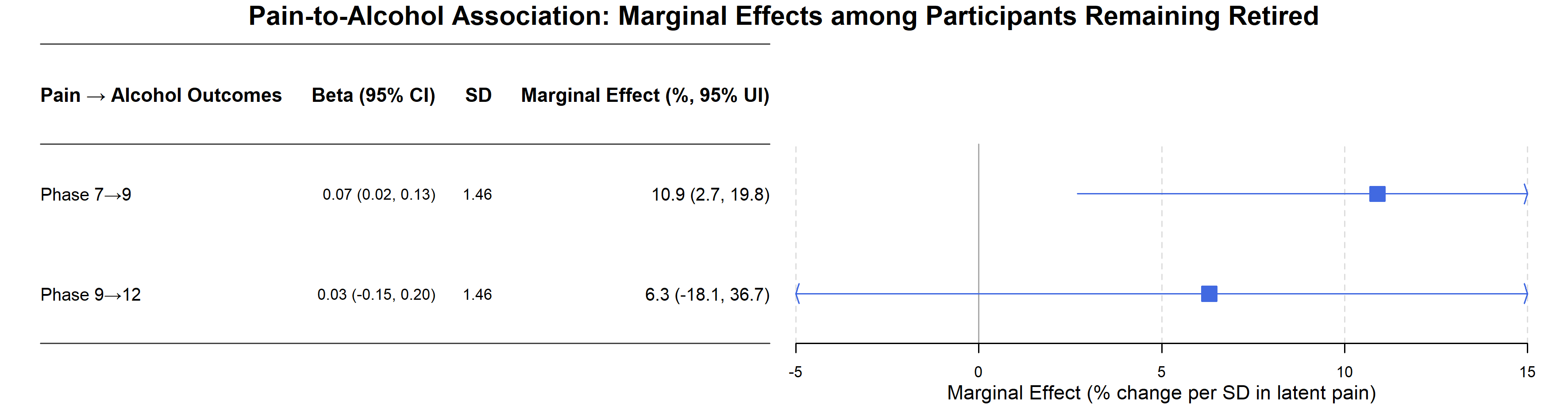


Different beta and 95% CI across three phases: we acquired the standardized beta from the fully-adjusted RI-CLPM without equality constraints.

SD: every standard deviation of continuous latent response variables for overall pain severity at two prior phases (phases 7 and 9).

**Figure S9**. Marginal effect of probability increase of no-, mild-, and severe-pain levels in subsequent phases per every standard deviation of pure alcohol consumption at phases 7 and 9 among participants experiencing retirement transition.


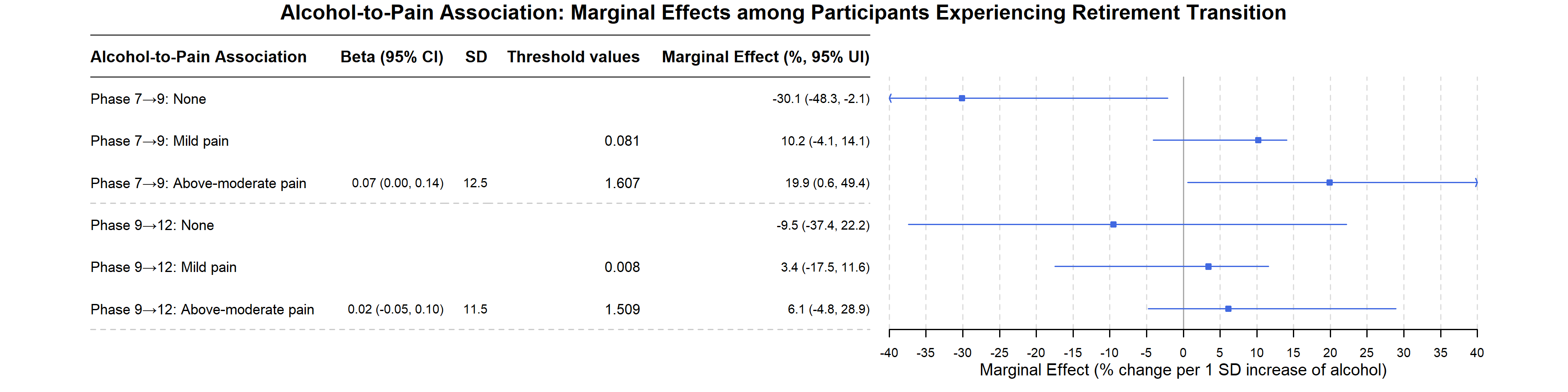


Different beta and 95% CI across three phases: we acquired the standardized beta from the fully-adjusted RI-CLPM without equality constraints.

SD: every standard deviation of pure alcohol units at two prior phases (phases 7 and 9).

Threshold values: threshold values for mild- and above-moderate-pain level at two subsequent phases (phases 9 and 12).

The 95% uncertainty intervals for marginal effects were computed through Monto-Carlo simulation with 1000 draws.

**Figure S10**. Marginal effect of probability increase of no-, mild-, and above-moderate-pain levels in subsequent phases per every standard deviation of pure alcohol consumption at phases 7 and 9 among midlife participants experiencing retirement transition.


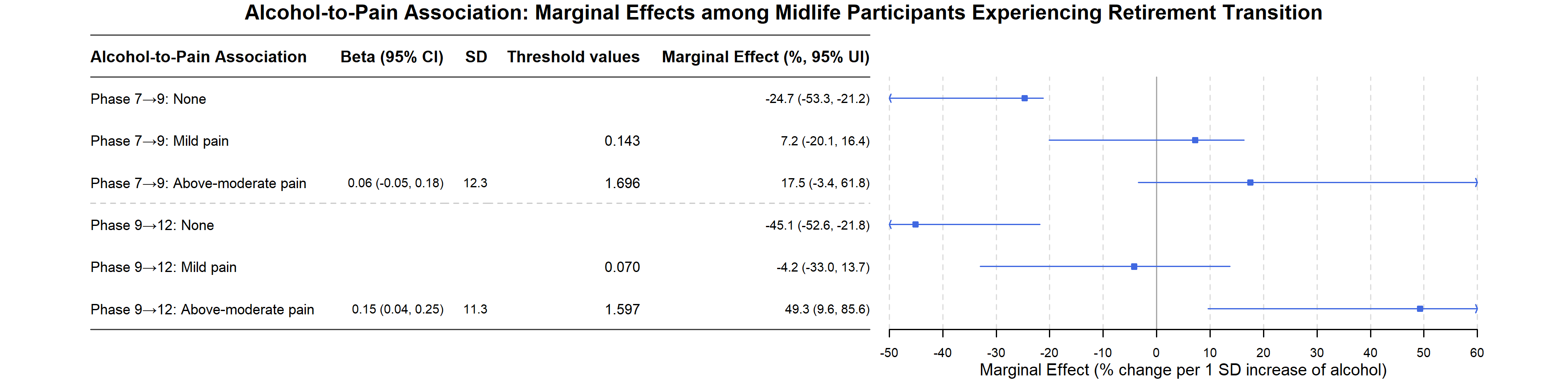


Different beta and 95% CI across three phases: we acquired the standardized beta from the fully-adjusted RI-CLPM without equality constraints.

SD: every standard deviation of pure alcohol units at two prior phases (phases 7 and 9).

Threshold values: threshold values for mild- and above-moderate-pain level at two subsequent phases (phases 9 and 12).

The 95% uncertainty intervals for marginal effects were computed through Monto-Carlo simulation with 1000 draws.

**Figure S11**. Marginal effect of probability increase of no-, mild-, and above-moderate-pain levels in subsequent phases per every standard deviation of pure alcohol consumption at phases 7 and 9 among low-SEP participants.


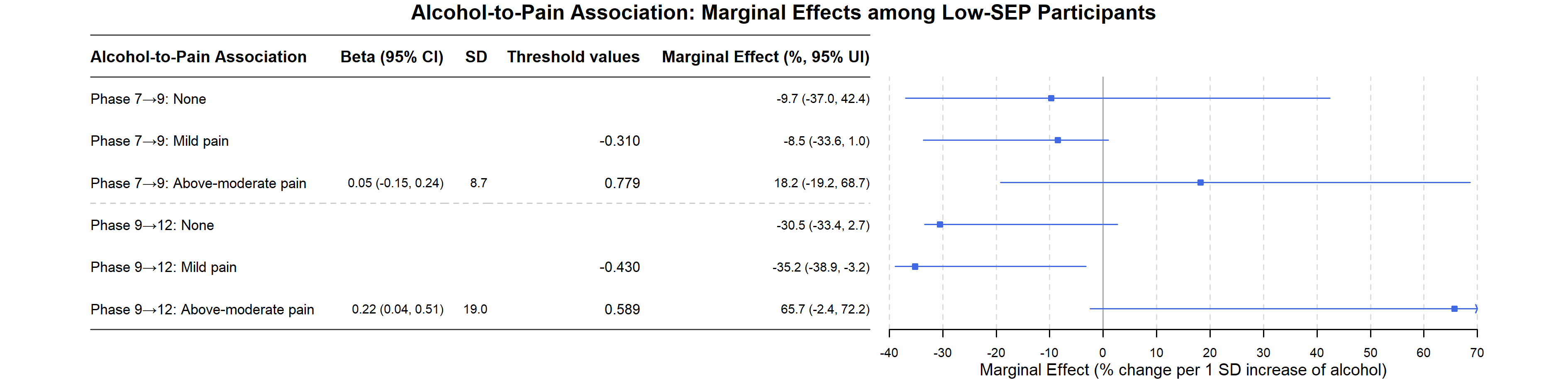


Different beta and 95% CI across three phases: we acquired the standardized beta from the fully-adjusted RI-CLPM without equality constraints.

SD: every standard deviation of pure alcohol units at two prior phases (phases 7 and 9). In this case, the larger SD of pure alcohol units at phase 9 may be attributed to the smaller sample size at phase 9 due to attrition.

Threshold values: threshold values for mild- and above-moderate-pain level at two subsequent phases (phases 9 and 12).

The 95% uncertainty intervals for marginal effects were computed through Monto-Carlo simulation with 1000 draws.

**Figure S12**. Marginal effect of probability increase of no-, mild-, and above-moderate-pain levels in subsequent phases per every standard deviation of pure alcohol consumption at phases 7 and 9 among high-SEP participants.


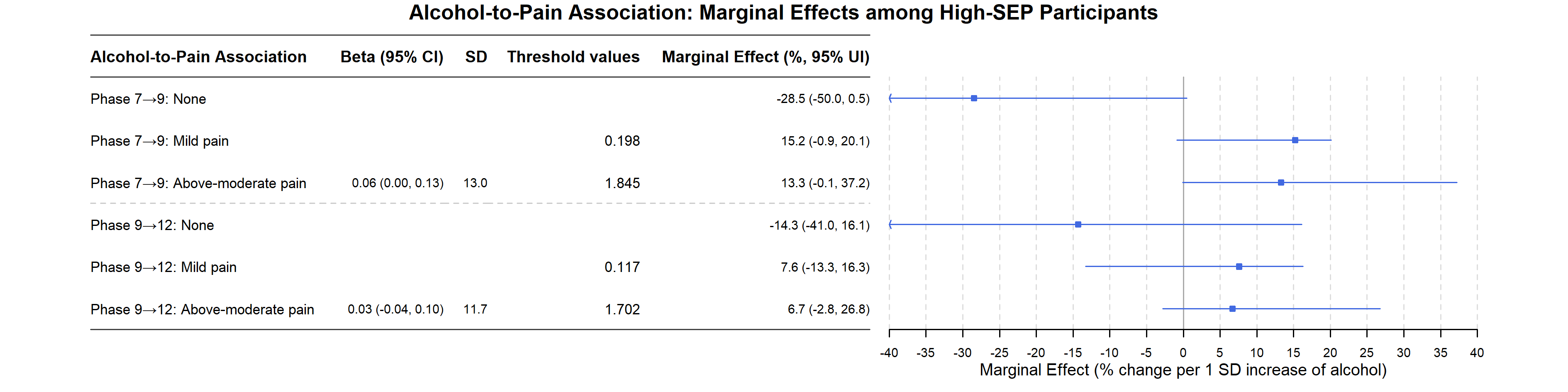


Different beta and 95% CI across three phases: we acquired the standardized beta from the fully-adjusted RI-CLPM without equality constraints.

SD: every standard deviation of pure alcohol units at two prior phases (phases 7 and 9).

Threshold values: threshold values for mild- and above-moderate-pain level at two subsequent phases (phases 9 and 12).

The 95% uncertainty intervals for marginal effects were computed through Monto-Carlo simulation with 1000 draw.
